# Organelle-mimicking liposome dissociates G-quadruplexes and facilitates transcription

**DOI:** 10.1093/nar/gku998

**Published:** 2014-10-21

**Authors:** Smritimoy Pramanik, Hisae Tateishi-Karimata, Naoki Sugimoto

**Affiliations:** 1 Frontier Institute for Biomolecular Engineering Research (FIBER), Konan University, 7–1–20 Minatojima-minamimachi, Chuo-ku, Kobe 650–0047, Japan; 2 Graduate School of Frontiers of Innovative Research in Science and Technology (FIRST), Konan University, 7–1–20 Minatojima-minamimachi, Chuo-ku, Kobe 650–0047, Japan

## Abstract

Important biological reactions involving nucleic acids occur near the surface of membranes such as the nuclear membrane (NM) and rough endoplasmic reticulum (ER); however, the interactions between biomembranes and nucleic acids are poorly understood. We report here that transcription was facilitated in solution with liposomes, which mimic a biomembrane surface, relative to the reaction in a homogeneous aqueous solution when the template was able to form a G-quadruplex. The G-quadruplex is known to be an inhibitor of transcription, but the stability of the G-quadruplex was decreased at the liposome surface because of unfavourable enthalpy. The destabilization of the G-quadruplex was greater at the surface of NM- and ER-mimicking liposomes than at the surfaces of liposomes designed to mimic other organelles. Thermodynamic analyses revealed that the G-rich oligonucleotides adopted an extended structure at the liposome surface, whereas in solution the compact G-quadruplex was formed. Our data suggest that changes in structure and stability of nucleic acids regulate biological reactions at membrane surfaces.

## INTRODUCTION

Biomembranes play pivotal roles in not only the cell structure but also various intracellular functions. For example, the nuclear membrane (NM) in eukaryotic cells is a lipid bilayer that surrounds the genomic DNA and associated components. The NM serves as a physical boundary and may also be involved in chromatin function and gene expression (1). Liposomes, simple artificial systems that mimic biomembranes (2), have been used to study the dynamics and structural features of many cellular processes (3). For example, it was recently reported that DNA undergoes a conformational transition from a folded state in the aqueous phase to a coiled state on the phospholipid membrane in a cell-sized microdroplet and that the conformational transition regulated transcriptional activity (4). Self-replication of DNA is observed within a self-reproducible cationic giant vesicle that serves as a model protocell (5). Moreover, the efficiency of *in vitro* gene expression is enhanced in the presence of liposomes (6–8). It has been reported that the antimicrobial peptide mastoparan X undergoes a coil-to-helix transition upon binding to membranes (9). Liposomes have been used to reproduce membrane fusion (10) and ion channel formation (11) using purified proteins reconstituted in the liposomes.

**Figure 1.**
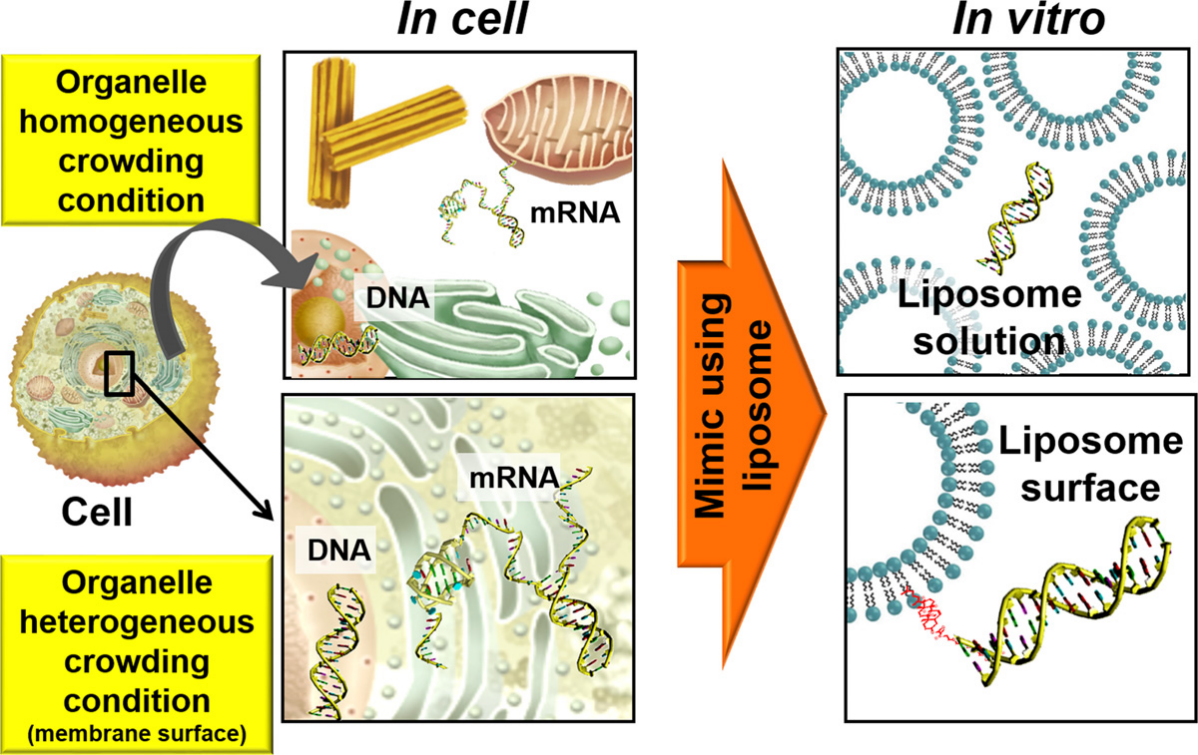
Schematic representation of intracellular crowding within organelles and heterogeneous conditions at the membrane surface. In this study, these intracellular conditions were mimicked using liposomes.

In living cells, biomembranes of organelles separate certain biomolecules from the rest of the cellular environment and create two kinds of environments (12). Inside organelles, such as nucleus, endoplasmic reticulum (ER) and mitochondria, high concentrations of biomolecules result in homogeneous crowding conditions (Figure 1). At the biomembrane surface, conditions are heterogeneous (Figure 1). Although the canonical structure of genomic DNA is a duplex, regions of DNA can undergo structural transitions from the duplex structure to non-canonical structures, such as G-quadruplexes, in response to environmental conditions (13–16). The formation of G-quadruplexes inhibits biological reactions, such as telomere elongation and transcription (17,18). To better predict whether G-quadruplexes form in cells, the structure and stability of the nucleic acids under conditions of molecular crowding induced by both non-interacting (19–22) and interacting (23) cosolutes have been studied. Formation of the G-quadruplexes is markedly facilitated by cosolutes (19). We have investigated the importance of heterogeneous confined media in the cell nucleus using reverse micelles and found that a significant fraction of the telomeric region of genomic DNA adopts non-canonical structures under these conditions (24). We have also recently shown that the formation of G-quadruplexes in open reading frames suppresses the translation of mRNA into protein (25). Although most proteins are translated on ribosomes that are free in the cytoplasm, translation of membrane proteins occurs on ribosomes bound to the membrane surface (12). The structures of mRNA on these membrane-bound ribosomes may be affected by the heterogeneous conditions at the membrane surface, in turn affecting translation efficiency.

**Figure 2.**
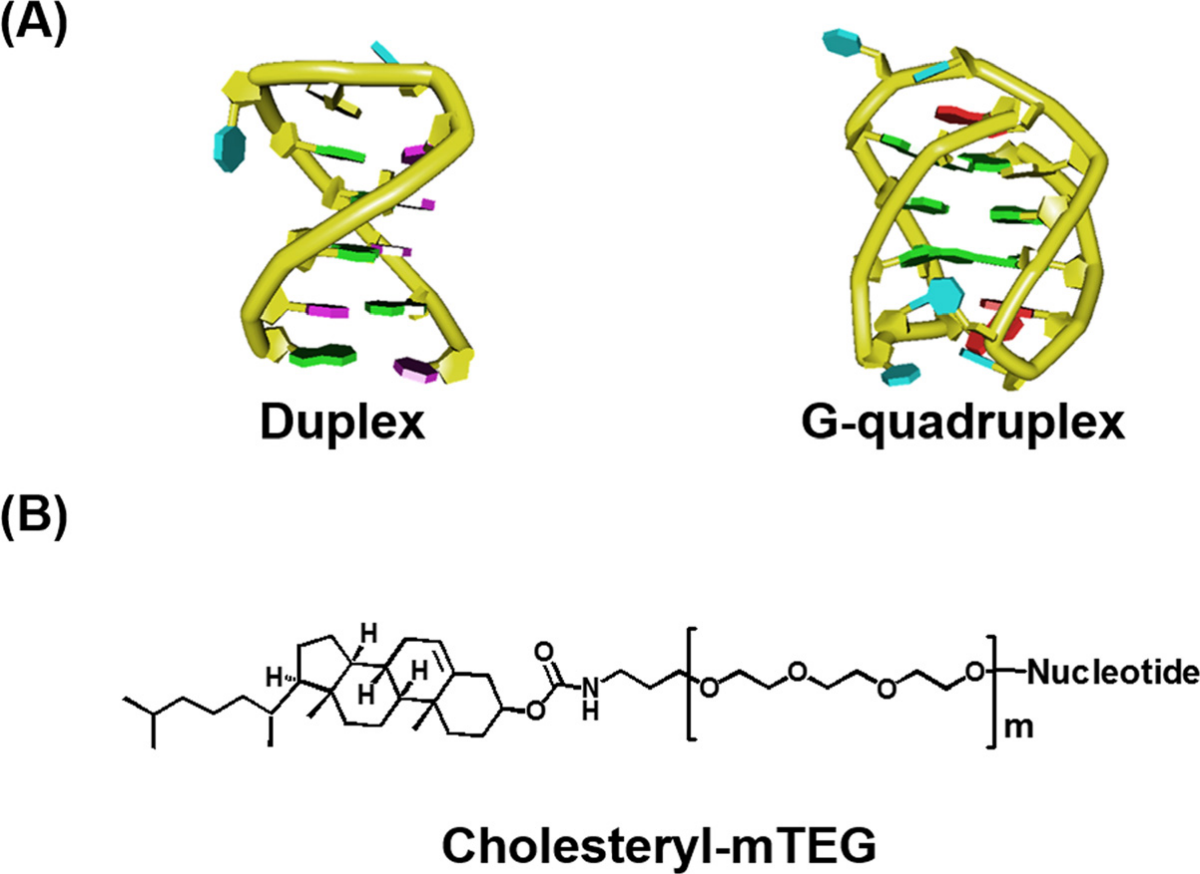
(**A**) Schematic representations of DNA duplex and G-quadruplex. (**B**) Chemical structure of cholesteryl-mTEG (*m* = 1, 2, 3 and 4), which was used as 5′ end modification for oligonucleotides.

**Figure 3.**
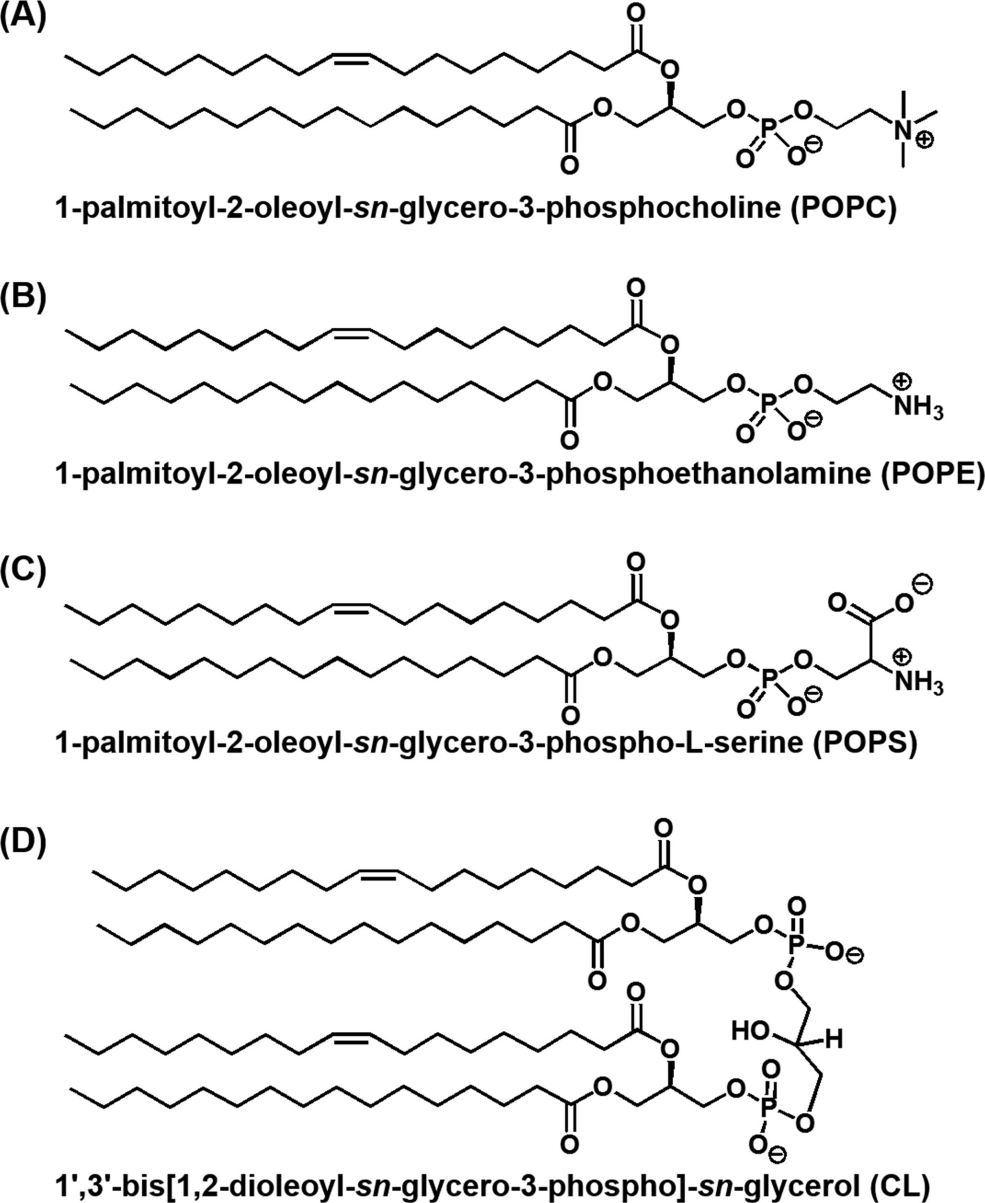
Chemical structure of (**A**) POPC, (**B**) POPE, (**C**) POPS and (**D**) CL.

In the present study, we investigated the structure and stability of DNA hairpins and DNA and RNA G-quadruplexes in solutions containing liposomes to mimic the crowded condition present inside organelles and at liposome surfaces, which mimic the heterogeneous conditions at the biomembrane surface (Figure 1). The sequences of the DNA oligonucleotides we studied are 5′-GGAAGCTTTTTGCTTCC-3′ (*D*; the loop region is underlined), which is able to form a hairpin with a stem of six base pairs, and the human telomeric DNA sequence 5′-TAG_n_TTAG_n_TTAG_n_TTAG_n_-3′ (*G_n_*: *n* = 2, 3 and 4; the loop regions are underlined), which is able to form an intramolecular G-quadruplex (Figure 2 and Supplementary Figure S1). To mimic cellular organelle membranes we used different liposome preparations. We used 1-palmitoyl-2-oleoyl-*sn*-glycero-3-phosphocholine (POPC), 1-palmitoyl-2-oleoyl-*sn*-glycero-3-phosphoethanolamine (POPE), 1-palmitoyl-2-oleoyl-*sn*-glycero-3-phospho-L-serine (POPS) and 1′,3′-bis[1,2-dioleoyl-*sn*-glycero-3-phospho]-*sn*-glycerol (CL) at specific molar ratios to prepare biomimetics of the NM, rough ER and inner mitochondrial membrane (IM) (Figure 3 and Table 1) (26). NM and ER are of interest because nucleic acids, such as DNA and mRNA, are attached to these organelle surfaces (12). The IM is not known to directly interact with nucleic acids (12). Because cholesterol-based anchoring molecules are also found in eukaryotic membranes and these molecules can be incorporated into lipid membranes without disrupting the bilayer structure (26), we modified the 5′ ends of certain DNA oligonucleotides with different numbers of the cholesteryl-triethylene glycol (TEG) spacers (*mcD* and *mcG_n_*: *m* = 1, 2, 3 and 4) to induce binding to the liposome surface (Figure 2). To evaluate the effect of liposomes on the RNA G-quadruplex, we also studied a cholesterol-modified RNA oligonucleotide with a TEG spacer, 5′-cholesteryl-TEG-UAG_3_UUAG_3_UUAG_3_UUAG_3–_3′ (1crG_3_), which may adopt an intramolecular G-quadruplex structure.

**Table 1.** Lipid compositions of biomimetic liposomes^a^

Biomimetic liposome	Molar ratios of phospholipids (%)			
	POPC	POPE	POPS	CL
Nuclear membrane (NM)	69	25	6	−
Rough endoplasmic reticulm membrane (ER)	75	20	4	1
Inner mitochondrial membrane (IM)	43	39	1	17

^a^Vance, D. E.; Vance, J. E. *Biochemistry of Lipids, Lipoproteins and Membranes*; 4th ed, Elsevier, Amsterdam.

## MATERIALS AND METHODS

### Oligonucleotides and buffers

Oligonucleotides of high performance liquid chromatography purification grade were purchased from Japan Bio Service. Single-strand concentrations of oligonucleotides were determined by measuring the absorbance at 260 nm at a high temperature using a Simadzu 1700 spectrophotometer connected to a thermoprogrammer. Extinction coefficients were calculated from mononucleotide and dinucleotide data using the nearest-neighbour approximation (27). POPC, POPE, POPS and CL were purchased from Avanti Polar Lipids. All other chemicals, such as NaCl and Na_2_EDTA, were purchased from Wako and used as received.

### Preparation of liposomes

Liposomes were prepared using the procedure described elsewhere (28,29). Briefly, lipids were dissolved in chloroform/methanol solvent mixture (2:1). Under reduced pressure, lipid films were produced in a round-bottom flask by rotary evaporation. The lipid films were reconstituted and re-evaporated twice. The thin lipid film was kept under a high vacuum for 12 h. Dry N_2_ was blown over the film for 1 h to ensure that all organic solvents were removed. The lipid film was hydrated in a buffered solution containing 10 mM sodium phosphate (pH 7.0), 10 mM NaCl and 1 mM Na_2_EDTA. Six freeze-thaw cycles were performed to convert small vesicles into large multi-lamellar vesicles and then the solution was extruded 10 times thorough polycarbonate membrane filters with 100-nm pore diameter to prepare small unilamellar vesicles.

### Dynamic light scattering (DLS)

Measurements of the size of liposomes were made by DLS at 25°C using a Malvern Zetasizer Nano ZSP spectrometer with a detection range of 0.3−10000 nm. A 10-mW He-Ne laser with a beam wavelength of 633 nm was used as a light source. Data were obtained and processed via the Zetasizer v7.10 software. The mean hydrodynamic diameter of each liposome was obtained from seven independent measurements over a lipid concentration range of 0.25 to 1.0 mM. Before measurements, samples were filtered through polycarbonate membrane filters with 450-nm pore diameter.

### Differential scanning calorimetry (DSC)

DSC thermograms were obtained by using a MicroCal VP-DSC calorimeter with a cell volume of 0.5156 ml. Samples were prepared in buffer and filtered through polycarbonate membrane filters with 450-nm pore diameter. DSC measurements were performed from 1°C to 60°C. Prior to each scan, 2.0 mM liposomes were equilibrated at 1°C for 15 min. At least three calorimetric scans were performed for each sample with a heating rate of 1°C min^−1^. VP-DSC calorimeter was controlled via the VPViewer 2000 DSC software, and obtained data were analysed by using the Microcal Origin 5.0 software package. Reproducibility was high.

### Circular dichroism (CD) measurements

CD experiments were performed on a JASCO J-820 spectropolarimeter using a 1.0-mm path length cuvette at a total strand concentration of 20 μM. The CD spectra shown in this study are the averages of at least three scans measured from 220 to 350 nm at a scan rate of 50 nm min^−1^. The temperature of the cell holder was regulated by a JASCO PTC-348 temperature controller, and the cuvette-holding chamber was flushed with a constant stream of dry N_2_ gas to avoid condensation of water on the cuvette exterior. Before measurement, the sample was heated to 90°C, cooled at a rate of 1°C min^−1^ and incubated at 0°C for 1 h. Thermal denaturation was measured both in the dilute and liposome conditions at indicated wavelengths. The heating rates were 0.5°C min^−1^. It is important to note that under all the conditions, denaturation and renaturation profiles of 1cG_3_ were identical as assessed by measuring CD intensity at 295 nm. This lack of hysteresis and the presence of an isodichroic point confirmed that the transition between single strand and G-quadruplex was two state.

### Transcription assays

Transcription reactions were carried out at 37°C in a total volume of 20 μl. T7 RNA polymerase was present at 0.3 μM. The DNA templates used were 5′-cholesteryl-TEG-GGGGTTAGGGGTTAGGGGTTAGGGGTTGTAACTATCGGGTGTGTAGTTCGTGTCATCTCCTATAGTGAGTCGTATTAGTGATC-3′ (containing a G-quadruplex forming region) and 5′-cholesteryl-TEG-GGTGTATTGTAACTATCGAGGCAGAGAGAGCACCGAGCCTAGTTCGTGTCATCTCCTATAGTGAGTCGTATTAGTGATC-3′ (control without G-quadruplex forming region). The T7 promoter regions are underlined. The template concentration in reactions was 1.5 μM. After incubating at 37°C for 10 min, ribonucleoside triphosphate (NTPs) were added to a final concentration of 1 mM each to initiate the reaction. The final reaction buffer contained 30 mM KCl, 40 mM Tris-HCl (pH 8.0 at 37°C), 8 mM MgCl_2_, 2 mM spermidine and 5 mM dithiothreitol (DTT). Reactions were quenched after incubation at the time indicated by addition of DNase I. After incubation for 10 min, a 20-fold excess volume of transcription stop solution (80 wt% formamide, 10 mM Na_2_EDTA and 0.01% blue dextran) was added. The samples were then heated to 90°C for 5 min, cooled rapidly and loaded onto a 10% polyacrylamide, 7 M urea gel. After electrophoresis at 60°C, the gels were stained by SYBR Gold (PerkinElmer Life Sciences), and levels were quantified with a fluorescent imager (FUJIFILM, FLA-5100).

## RESULTS

### Characterization of organelle-mimicking liposomes

Unilamellar organelle-mimicking liposomes were prepared and characterized by DLS and DSC. The compositions of the liposomes are listed in Table 1. The mean hydrodynamic diameters of POPC, IM, NM and ER liposomes were 127 ± 1.86, 125 ± 1.74, 122 ± 1.71 and 122 ± 1.68 nm, respectively (Supplementary Figure S2). Narrow size distributions were obtained for each liposome (Supplementary Figure S2). Lipid packing at the liposome bilayer was studied using DSC. Liposomes prepared from synthetic phospholipids undergo transitions at well-defined temperatures based on their structure, lipid composition and the physicochemical properties of the solvent in which they are dispersed (30–32). The phase transition of pure POPC liposomes occurs at −2.5 ± 2.4°C (31,33,34). To validate our DSC experimental procedure, we analysed pure 1,2-dipalmitoyl-*sn*-glycero-3-phosphocholine (DPPC) liposomes, which have a gel to liquid-crystalline phase transition (41.3 ± 1.8°C) at a temperature well above the ice-water transition (31,35,36). In agreement with previous reports, the temperature and enthalpy of the gel to liquid-crystalline phase transition for DPPC liposomes were estimated to be 40.5 ± 0.1°C and +8.2 ± 0.1 kcal mol^−1^, respectively (31,35,36). DSC themograms of ER, NM and IM liposomes were different from that of pure POPC liposomes, although we could not trace the main phase transition peaks of POPC, ER, NM and IM liposomes (Supplementary Figure S3). Moreover, DSC themograms of ER, NM and IM liposomes differed from each other. These results suggest that POPC, POPE, POPS and CL lipids associated to form heterogeneous liposomes with uniform sizes that mimic various organelles.

### Effect of organelle-mimicking liposomes on the structure and stability of DNA duplexes and G-quadruplexes

The structures of oligonucleotides were studied by CD spectroscopy in both the absence and presence of liposomes. We used a buffer containing 10 mM sodium phosphate (pH 7.0), 10 mM NaCl and 1 mM Na_2_EDTA in all experiments. The duplex-forming sequence D folded into the canonical intramolecular anti-parallel-stranded duplex with a positive peak at ∼282 nm and a negative peak at ∼255 nm in the absence of liposomes (Supplementary Figure S4A) (37–39), and the CD spectrum was unaltered in the presence of liposomes (Supplementary Figure S4A). Because the human telomere sequence is a repeating element of TTAGGG that has been studied extensively (17,18,24), we investigated the structure and stability of G_3_ in the absence and presence of liposomes. The CD spectrum of 20 μM of G_3_ in the absence of liposomes showed a positive peak at ∼295 nm and a negative peak at ∼260 nm, indicating the formation of a basket-type, anti-parallel G-quadruplex structure (Supplementary Figure S4B) (37–39). In the presence of 2.0 mM liposomes of any kind, the CD signatures of G_3_ were also unaltered compared with that in the absence of liposomes, indicating that the liposomes we studied induced no structural changes in the oligonucleotides (Supplementary Figure S4B). The CD spectra of G_2_ and G_4_ were also characteristic of anti-parallel G-quadruplex structures in both the absence and presence of the liposome formulations (Supplementary Figure S5).

The CD intensity of oligonucleotides was recorded as a function of temperature to investigate the stability of the structures formed by the oligonucleotides in different conditions. For D, the thermal stability was the same within experimental error in the absence and presence of 2.0 mM IM, NM and ER liposomes (*T*_m_, 59.5°C, Supplementary Figure S4C). The thermal stabilities for G_2_, G_3_ and G_4_ were also the same in the absence and presence of 2.0 mM IM, NM and ER liposomes (Supplementary Figures S4D, S5C and S5D). We concluded that crowding conditions induced by the organelle-mimicking liposomes had no effect on structures or stabilities of canonical and non-canonical DNA structures. It has been reported that molecular crowding decreases the stability of a duplex and increases that of the G-quadruplex by decreasing water activity (19–22). The water activities of solution without liposome and with 2.0 mM IM, NM or ER liposomes were not significantly different (*a*_w_ = 0.993, where *a*_w_ is water activity estimated by vapour pressure osmometry). This likely explains why these liposome solutions had no effect on stabilities of structures adopted by the DNA oligonucleotides studied here.

### Effect of heterogeneous conditions on membrane surfaces on the structures and stabilities of duplex and G-quadruplexes

**Figure 4.**
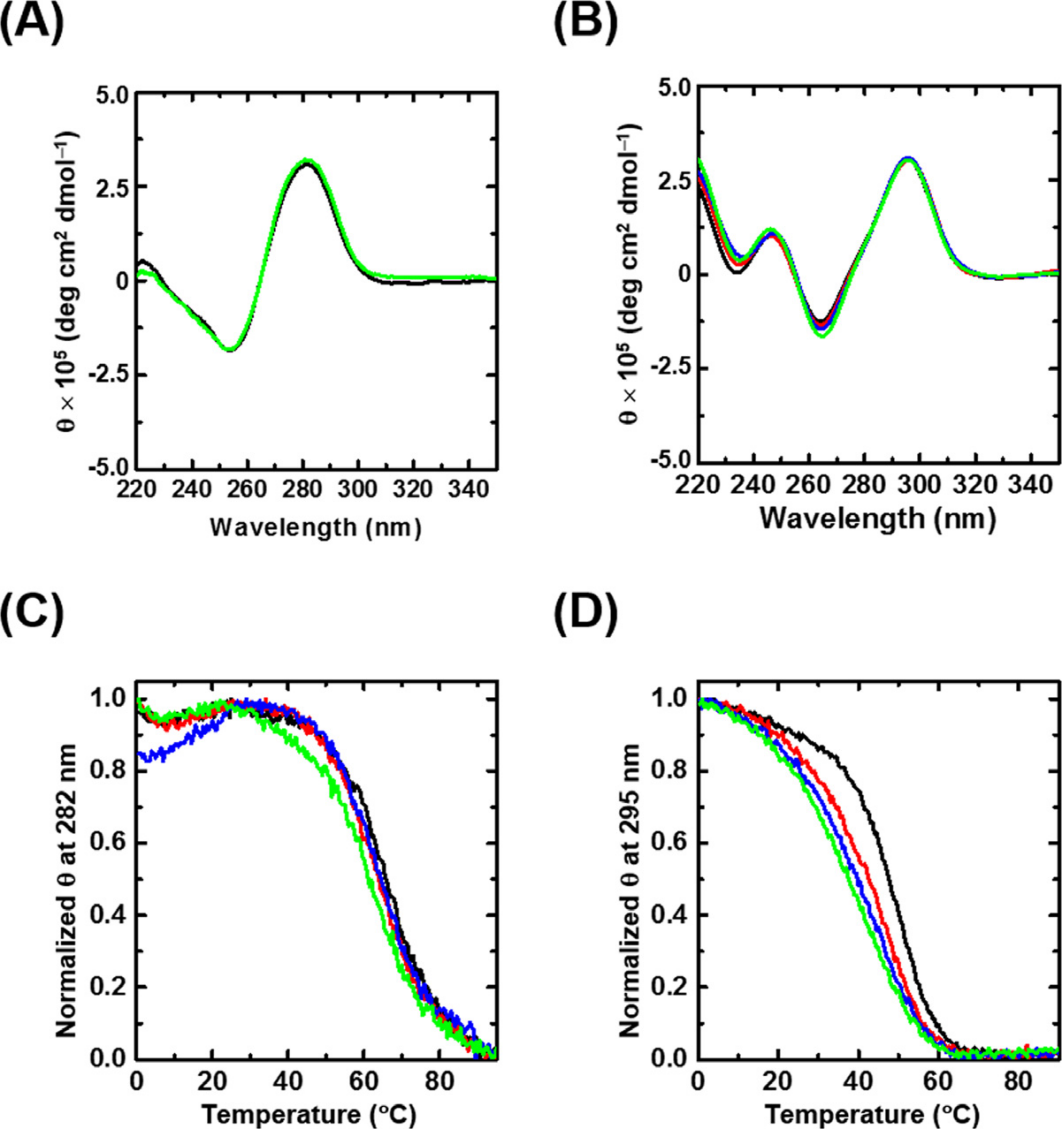
CD spectra of 20 μM (**A**) 1cD and (**B**) 1cG_3_ at 4°C and normalized CD melting profiles of (**C**) 1cD at 282 nm and (**D**) 1cG_3_ at 295 nm in the absence (black) and presence of 2.0 mM IM membrane (red), NM (blue) and rough ER (green) liposomes.

The CD signatures of the cholesterol-conjugated 1cD (Figure 4A) and 1cG_3_ (Figure 4B) were similar to those of D and G_3_, respectively, in both the absence and presence of 2.0 mM liposomes of all kinds. 1cG_2_ and 1cG_4_ also formed anti-parallel G-quadruplexes in both the absence and presence of liposomes (Supplementary Figure S6A and SB). The thermal stability of 1cD slightly decreased in the presence of 2.0 mM biomimetic liposome compared with that in the absence of any liposome (Figure 4C). The extent of destabilization caused by liposomes differed. In the absence of liposomes, the *T*_m_ was estimated to be 65.5°C (Figure 4C). In the presence of 2.0 mM IM, NM and ER, the *T*_m_s were 64.5°C, 64.5°C and 62.5°C, respectively. The thermal stability of 1cG_3_ significantly decreased in the presence of 2.0 mM biomimetic liposomes compared with that in the absence of any liposome (Figure 4D). For 1cG_3_, *T*_m_ was estimated to be 50.0°C in the absence of liposomes, whereas the *T*_m_'s were 47.0°C, 45.0°C and 42.5 °C in the presence of 2.0 mM IM, NM and ER liposomes, respectively. These data indicate that the thermal stabilities of 1cD and 1cG_3_ depend on the lipid composition of biomembranes. The thermal stabilities for 1cG_2_ and 1cG_4_ also decreased in the presence of biomimetic liposomes (Supplementary Figure S6C and S6D). NM and ER membranes are known to interact with nucleic acids and the liposome mimics of these organelles markedly destabilized the DNAs. Therefore, heterogeneous conditions at organelle surfaces could play a key role in the stability of nucleic acid structures.

To determine how the lipid composition of biomembranes altered the stabilities of nucleic acid structures, we evaluated the extent of destabilization (Δ*T*_m_). Δ*T*_m_ was calculated by subtracting the *T*_m_ of the nucleic acid structure in the presence of liposomes from the *T*_m_ in the absence of 2.0 mM liposomes. Δ*T*_m_s for 1cD duplexes were 1.0°C, 1.0°C and 3.0°C in IM, NM and ER liposomes, respectively. Δ*T*_m_s for the 1cG_3_ G-quadruplexes in the presence of IM, NM and ER liposomes were 3.0°C, 5.0°C and 7.5°C, respectively. Therefore, the ER liposome was the most destabilizing of the formulations evaluated for both duplexes and G-quadruplexes, and the extent of destabilization caused by ER liposome was higher for G-quadruplexes than for duplexes. Δ*T*_m_ increased with the amount of POPC in biomimetic liposomes. In IM, NM and ER liposomes, the mole fraction of POPC was 43%, 69% and 75%, respectively, of the total lipid (Table 1). Therefore, POPC is probably the constituent of biomimetic liposomes that destabilizes the DNA structures.

### Effect of liposomes on thermodynamic parameters of the G-quadruplex formation

We next studied G-quadruplexes in pure POPC liposomes. POPC had no effect on the structure of the anti-parallel G-quadruplexes of 1cG_3_ (Supplementary Figure S7A). The thermal stability of the 1cG_3_ decreased when the concentration of POPC was increased from 0 to 2.0 mM (Supplementary Figure S8). An increase in temperature from 0°C to 90°C resulted in decreases and increases of the positive and negative peak intensities of 1cG_3_, respectively, with clear isodichroic points in the absence and presence of 2.0 mM POPC (Supplementary Figure S9). This finding indicates that the melting transition of 1cG_3_ was two state under the conditions studied.

We extracted thermodynamic parameters from melting curves as described previously (19–22). The value of Δ*G*°_25_ (free energy change of the formation of a G-quadruplex at 25°C) for 1cG_3_ increased from −3.1 to −1.2 kcal mol^−1^ when the concentration of POPC was increased from 0 to 2.0 mM (Table 2). The values of Δ*H*° and *T*Δ*S*° for the formation of the G-quadruplex also increased when the concentration of POPC was increased. Therefore, the 1cG_3_ G-quadruplex was destabilized by the liposome because of an unfavourable enthalpic contribution. We recently reported that choline ions bind to bases of single-stranded DNA, particularly unpaired guanines, and decrease duplex stability via an unfavourable enthalpic contribution (40). It is likely that the choline group at the liposome interface binds to the guanine bases of 1cG_3_, resulting in the observed decrease in the G-quadruplex stability in the presence of POPC. To test this hypothesis, we measured the structure and stability of 1cG_2_ and 1cG_4_ in the absence and presence of 2.0 mM POPC. The anti-parallel G-quadruplexes of both 1cG_2_ and 1cG_4_ (Supplementary Figure S7B and S7C) were destabilized in the presence of POPC liposomes (Supplementary Figure S10), again because of unfavourable enthalpic contributions (Supplementary Table S1).

**Table 2.** Thermodynamic parameters for formation of G-quadruplex by 1cG_3_^a^

POPC concentration (mM)	Δ*H*° (kcal mol^−1^)	*T*Δ*S*° (kcal mol^−1^)	Δ*G*° _25_ (kcal mol^−1^)	*T*_m_ (°C)
0.0	−40.0 ± 0.6	−36.9 ± 0.5	−3.1 ± 0.1	50.0
0.5	−30.7 ± 0.4	−28.4 ± 0.6	−2.3 ± 0.2	48.5
1.0	−23.3 ± 0.8	−21.9 ± 0.7	−1.4 ± 0.2	43.9
2.0	−22.1 ± 0.4	−20.9 ± 0.4	−1.2 ± 0.1	40.5

^a^All experiments were performed in buffers containing 10 mM sodium phosphate (pH 7.0), 10 mM NaCl, 1 mM Na_2_EDTA and various concentrations of POPC. Values are means ± standard deviations from three independent measurements. Melting temperatures were measured at a total strand concentration of 20 μM.

To evaluate the effect of the number of guanines on the G-quadruplex stabilities in the presence of liposome, we evaluated the differences in thermodynamic parameters [Δ*X* = (*X* of G-quadruplex formation in the presence of liposomes) − (*X* of G-quadruplex formation in the absence of liposomes); where *X* is Δ*H*°, *T*Δ*S*° or Δ*G*°_25_). In the presence of POPC liposomes, the ΔΔ*G*°_25_ values were estimated to be +0.5, +1.9 and +3.2 kcal mol^−1^ for 1cG*_2_*, 1cG_3_ and 1cG_4_, respectively (Supplementary Table S2). Therefore, the ΔΔ*G*°_25_ values increased with the number of guanines in the oligonucleotide. The values of ΔΔ*H*° and Δ(*T*Δ*S*°) for 1cG_n_ G-quadruplexes also increased when the concentration of POPC increased from 0 to 2.0 mM (Supplementary Table S2). This supports our hypothesis that the G-quadruplexes were destabilized at the liposome surface by the unfavourable binding of choline to guanine bases. We then investigated the effect of liposomes on the RNA G-quadruplex. We observed that in both the absence and presence of 2.0 mM POPC, 1crG_3_ had a positive peak near 265 nm and a negative peak near 240 nm (Supplementary Figure S11), the characteristic CD signature of a parallel G-quadruplex (37–39). Similarly to DNA, the RNA G-quadruplex (1crG_3_) was destabilized in the presence of POPC liposomes because of an unfavourable enthalpic contribution (Supplementary Figure S12 and Supplementary Table S3). For the RNA G-quadruplex, the changes in thermodynamic parameters, ΔΔ*G°*_25_ ΔΔ*H°* and Δ(*T*Δ*S°*), due to the presence of liposome were +3.7, +22.3 and +18.6 kcal mol^−1^, respectively (Supplementary Table S4). The extent of change of each thermodynamic parameter due to the presence of 2.0 mM POPC was higher for the RNA G-quadruplex (1crG_3_) than for the DNA G-quadruplex of the same sequence (1cG_3_). The choline group forms a hydrogen bond with the backbone of nucleic acids (41). The 2′-hydroxyl group in the ribose sugar of RNA may also bind to choline groups; this would explain the higher degree of destabilization of the RNA G-quadruplex compared to the DNA G-quadruplex in the presence of liposome.

As expected, the extent of destabilization of human telomeric G-quadruplex (1cG_3_) caused by ER, NM and IM biomimetic liposome differed (Supplementary Table S4). The values of ΔΔ*G*°_25_, ΔΔ*H*° and Δ(*T*Δ*S*°) for the 1cG_3_ G-quadruplex also increased with the concentration of POPC in biomimetic liposomes of IM, NM and ER (Supplementary Table S5). Because the hydrophobic mixed acyl chains of POPC, POPE, POPS and CL are the same, these lipids differ only in their polar head group regions (Figure 3). POPC has a tetraalkylammonium ion in the polar head group. At neutral pH, POPC, POPE, POPS and CL have 0, 0, −1 and −2 net head group charges, respectively. The negative charges of POPS and CL may be counter-balanced by excess Na^+^ ions present in the experimental solutions. The thermal stabilities for 1cG_3_ were almost the same in the absence and presence of 2.0 mM of POPS (data not shown), although we were unable to prepare pure liposomes of POPE or CL. Given that ΔΔ*G*°_25_, ΔΔ*H*° and Δ(*T*Δ*S*°) increased with increasing the concentration of POPC in biomimetic liposomes (Supplementary Table S5), we propose POPC to be the constituent of biomimetic liposomes that destabilizes the 1cG_3_ G-quadruplex.

### Effect of proximity to the liposome surface on G-quadruplex stability

**Figure 5.**
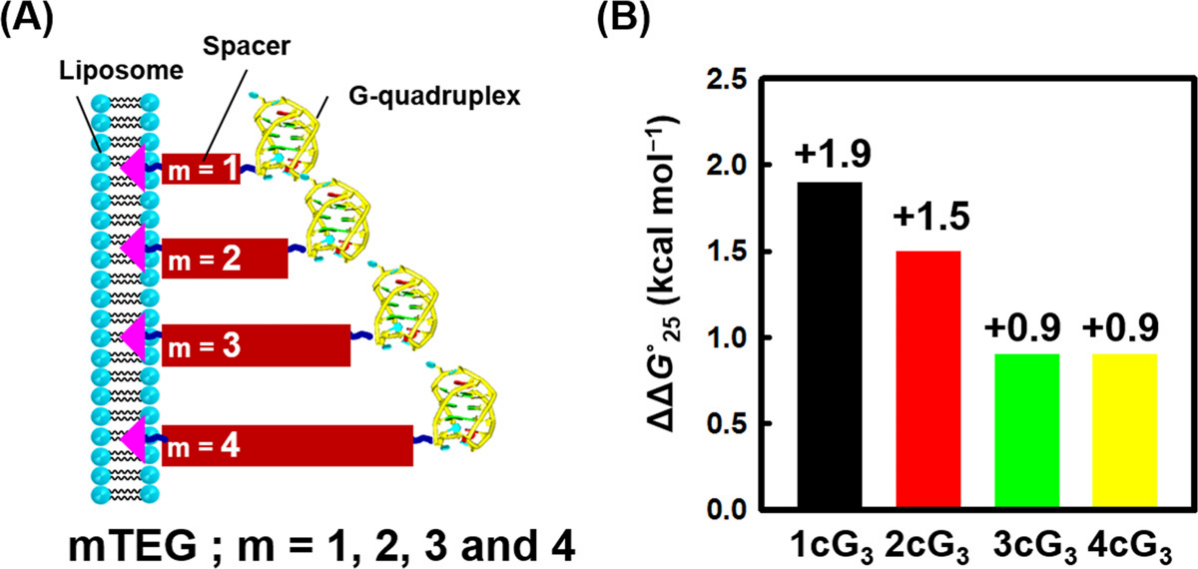
(**A**) Schematic representation of the location of G-quadruplexes at the liposome surface as a function of spacer TEG length (Scheme 2B). (**B**) ΔΔ*G*°_25_ in the presence of 2.0 mM POPC liposome for 1cG*_3_* (black), 2cG*_3_* (red), 3cG*_3_* (green) and 4cG*_3_* (yellow).

To evaluate the importance of proximity to the liposome surface on G-quadruplex stability, we incorporated different numbers of TEG spacers between the oligonucleotide and the cholesterol group that served to anchor the nucleic acid to the liposome surface. Oligonucleotides 2cG_3_, 3cG_3_ and 4cG_3_ have the same G-quadruplex-forming sequence but differ in the spacer length (Figure 5A). The CD signatures of 2cG_3_, 3cG_3_ and 4cG_3_ were identical in the absence and presence of 2.0 mM POPC, indicating that spacer length had no effect on oligonucleotide structure (Supplementary Figure S13). However, as the spacer length increased, the thermal stability decreased (Supplementary Table S1). In the presence of POPC, all were destabilized compared with their stability in the absence of liposomes (Supplementary Figure S14 and Supplementary Table S1). With increasing spacer length, which moved the structure further from the membrane surface, the values of the net free energy change (ΔΔ*G*°_25_) decreased (Figure 5B).

### Effect of liposomes on transcription reactions

**Figure 6.**
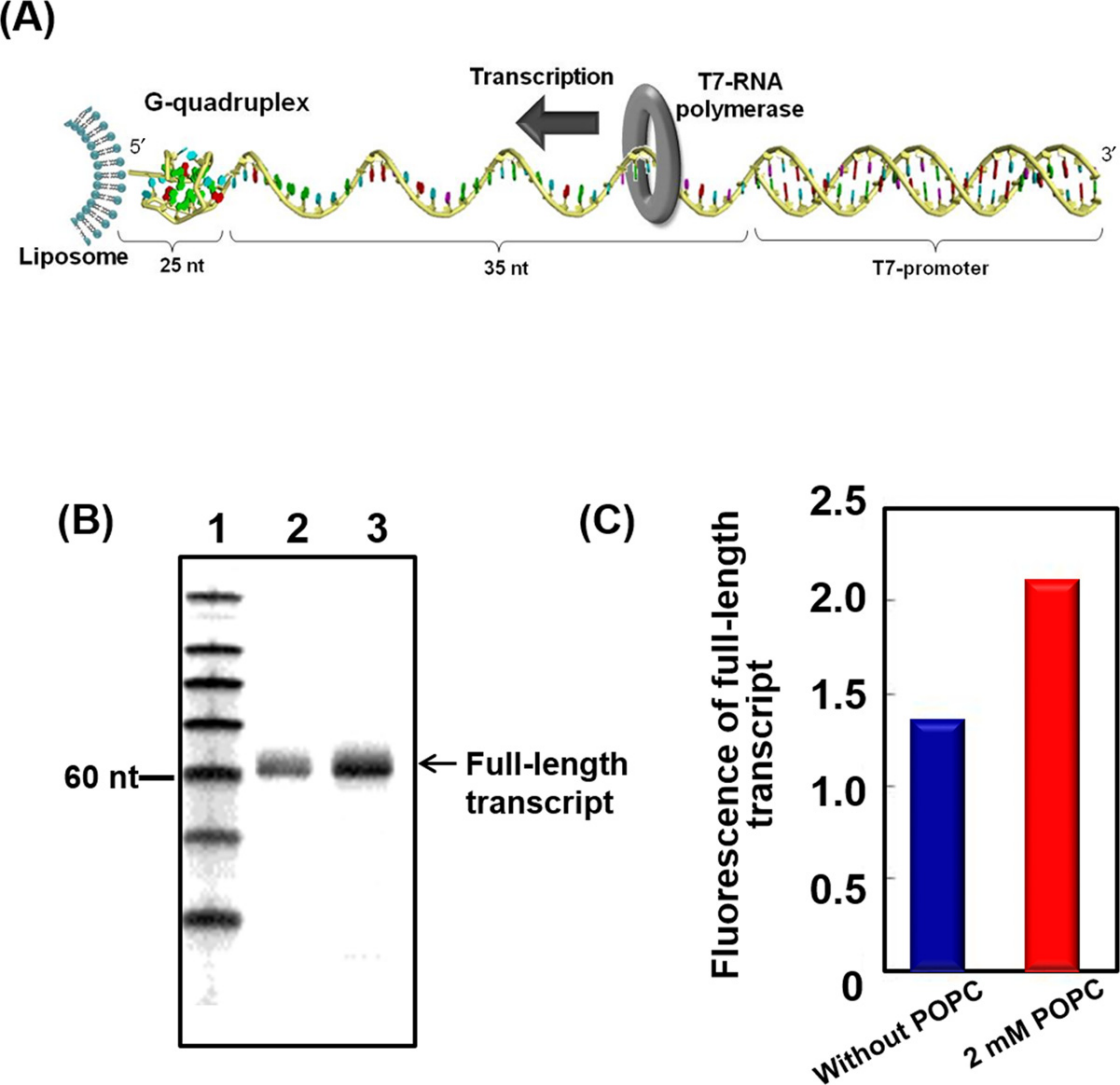
Effects of membrane surface on transcription of a template that contains a G-quadruplex-forming sequence. (**A**) Illustration of the template DNA immobilized on a liposome. (**B**) Denaturing gel electrophoresis of products of transcription reactions performed for 90 min at 37°C. Reaction mixtures contained 0.3 μM T7 polymerase and 1.5 μM DNA template in a buffer containing 10 mM NaCl, 40 mM Tris-HCl (pH 8.0), 8 mM MgCl_2_ and 2 mM spermidine in the absence and presence of 2.0 mM POPC. Lane 1, size marker; lane 2, transcription products in the absence of POPC; lane 3, transcription products in the presence of 2.0 mM POPC. (**C**) Comparison of fluorescence intensities of full-length transcripts from gel bands in Figure 6B (see Experimental Section for more detail).

To study the effect of the membrane surface on a process involving nucleic acids, we evaluated transcription. It was previously reported that the formation of non-canonical structures, such as a G-quadruplex, reduce transcription efficiency because these structure induce pausing of the polymerase (42,43). For a template, we used a cholesterol-conjugated DNA with a G-quadruplex forming region near the 5′ end (Figure 6A). T7 RNA polymerase transcription of this template under multi-turnover conditions was almost saturated at 90 min (data not shown). Figure 6B shows the results of gel electrophoretic analysis of transcription performed for 90 min at 37°C under multi-turnover conditions. In both the absence and presence of 2.0 mM POPC, transcription proceeded to the end of the DNA template, resulting in the formation of a full-length transcript of 60 nucleotides (Figure 6B, lanes 2 and 3). The amount of full-length transcript was increased by 56% in the solution containing 2.0 mM POPC compared with the solution in the absence of POPC (Figure 6C). As an additional control, we evaluated transcription of a cholesterol-conjugated template DNA that does not have a G-quadruplex forming region. The amount of full-length transcript from template DNA without any G-quadruplex forming region was increased in the presence of 2.0 mM POPC, compared with the solution in the absence of liposome (Supplementary Figure S15). However, the increment of transcript was much smaller (16%) than that from the template DNA with G-quadruplex forming region (56%). Together, our data indicate that membrane surface reduced G-quadruplex stability and facilitated transcription.

## DISCUSSION

### G-quadruplexes are destabilized by heterogeneous conditions at a membrane surface

Studies of nucleic acids in the presence of liposomes suggest that nucleic acid-membrane association is regulated electrostatic interactions with the lipid head groups (44). Based on studies of tRNA in the presence of lipids, it appears that hydrophobic interactions between nucleo-bases and hydrocarbon chains of phospholipids also affect adsorption of nucleic acids onto liposome surfaces (7,45–48). These previous studies provided qualitative explanations for nucleic acid-lipid interactions. Here we evaluated interactions of liposomes and nucleic acids from a thermodynamic point of view. Five factors play major roles in determining the stabilities and structures of nucleic acids: hydrogen bonding, base stacking, conformational entropy, solvation and cation binding (38). The combination of the free energy changes associated with each factor determines the overall stability of nucleic acids. It is difficult to quantitatively assess the contribution from each factor because free energy changes are measured for the overall process of the nucleic acid structure formation from a random coil state. With the exception of entropy loss, the factors mentioned favour nucleic acid structure formation. One of the important physical property differences between the liposome surface and bulk solution is a change in dielectric constant: The dielectric constant is 30–40 at the POPC liposome surface compared with 78 for water (7,49). Reductions in the dielectric constant of media destabilize duplex formation and favour G–quadruplex formation (50).

Here, however, we observed that proximity to a liposome surface destabilized the G-quadruplex structure, suggesting that forces in addition to the dielectric constant are important at the POPC liposome surface. Based on CD analysis, the structure adopted by the oligonucleotide was the same at the liposome surface as in buffer; therefore, destabilization was not due to a conformationally induced entropy change. Choline ions preferentially bind to guanine and cytosine relative to the other bases or the backbone through hydrogen bond formation and stabilize the single-stranded state relative to the duplex conformation (41). It is possible that guanine bases form specific hydrogen bonds with the choline head group of POPC lipid to enthalpically destabilize G-quadruplex formation at the liposome surface. This preferential binding of POPC head groups to guanines may also alter the solvation of the G-quadruplexes at the liposome surface compared with solvation in bulk solution. There was no change in water activity between the solution without liposomes and the solution containing 2.0 mM POPC liposomes; however, it is possible that hydrogen bond formation between single-stranded guanines and the POPC head group results in a different hydration level for the single-stranded state than for the G-quadruplex. Therefore, we suggest that the G-quadruplexes are destabilized at the liposome surface predominantly because of the preferential binding of the choline group head group of POPC to the guanine bases. The DNA duplex was less destabilized at the liposome surface than was the G-quadruplex probably because of the presence of fewer guanines in the DNA duplex forming oligonucleotide 1cD. We attempted to evaluate DNA duplex-forming oligonucleotides that contained more guanines, but the higher stability of such duplexes limited our ability to obtain quantitative parameters. Another reason for the higher destabilization of G-quadruplexes relative to duplexes may be that the average hydrogen bond energy in a Watson–Crick pair is 0.22 eV, whereas that in a G-quartet is 0.42 eV (51). Therefore, disruption of one G-quartet results in loss of more energy than the loss of a base pair in a duplex.

### Enthalpy–entropy compensation at a membrane surface

In the formation of nucleic acid structures, the standard enthalpy change from the initial state to the final state (Δ*H*°) is a quantitative measure of the changes in intermolecular bond energies that include hydrogen bonding and van der Waals interactions. The standard entropy change (Δ*S*°) reflects the degree of rearrangement during the process. Although there is not a linear relationship between enthalpy and entropy in equilibrium thermodynamics (52), enthalpy–entropy compensation phenomena have been observed in a wide variety of molecular events (53,54). The slope of *T*Δ*S*° versus Δ*H*° plot is the change of *T*Δ*S*° per unit change of enthalpy and is known as the dimensionless temperature unit (55,56). If the slope of the plot is less than unity, the favourable enthalpy change (negative Δ*H*°) is higher than the unfavourable entropy change (negative *T*Δ*S*°). In contrast, if the slope is larger than unity, the favourable enthalpy change is lower than the unfavourable entropy change. The intercept is the entropy contribution to the free energy (intrinsic stability) when Δ*H*° is zero. In the case of a positive intercept, the molecular association is stabilized even in the absence of enthalpic stabilization, whereas a negative intercept means that particular process requires enthalpic stabilization.

**Figure 7.**
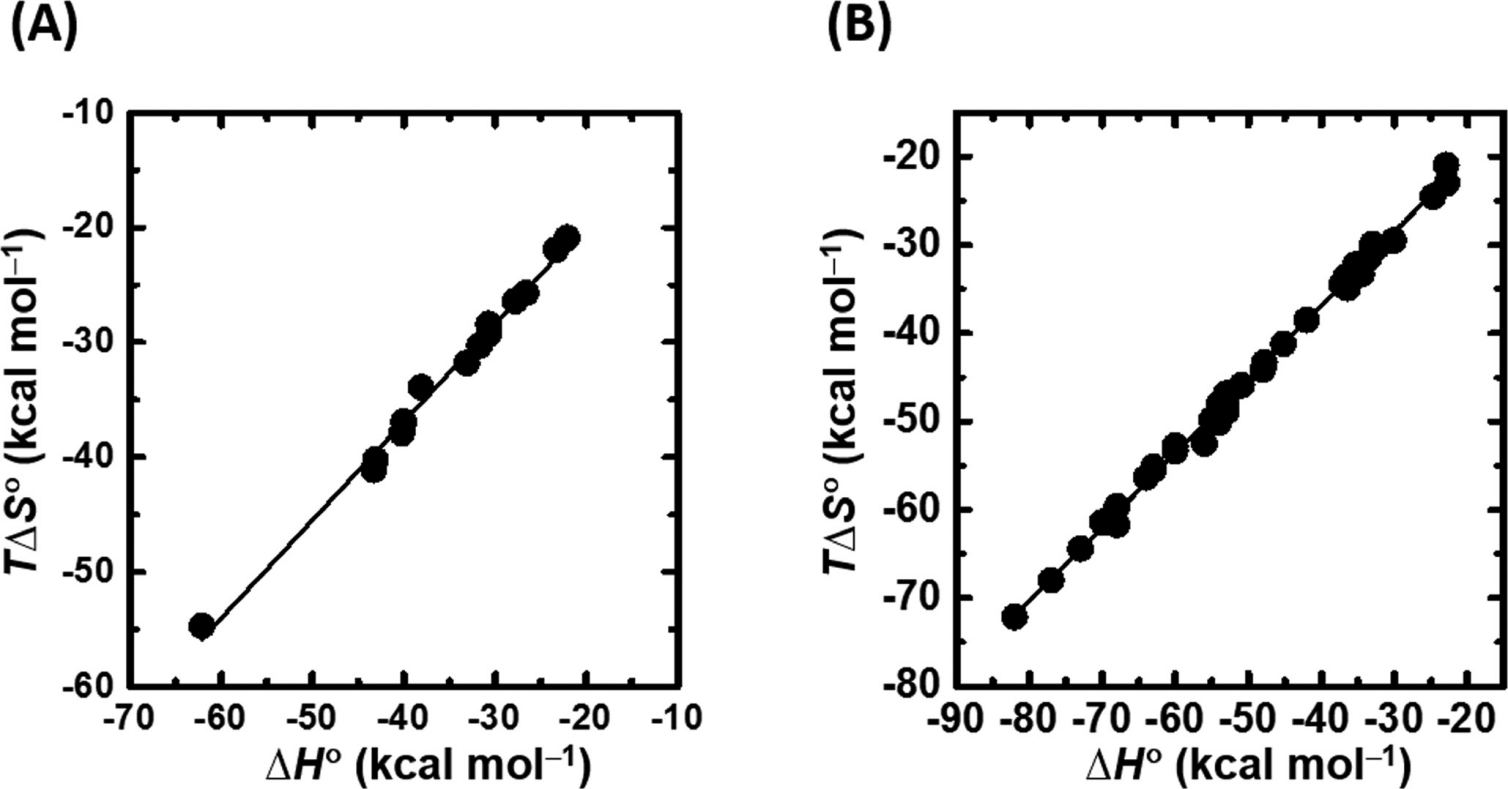
Enthalpy–entropy compensation plot for the folding thermodynamics of (**A**) *mcG_n_* G-quadruplexes in the presence of POPC liposome and (**B**) G-quadruplexes in the absence of liposome (18,42,43).

We summarize the slopes and intercepts of *T*Δ*S*° versus Δ*H*° plots for various molecular interactions characterized in this study in Table 2. In the presence of POPC liposomes, the plot of *T*Δ*S*° versus Δ*H*° for the *mcG_n_* G-quadruplexes reveals a strong linear correlation because of compensation between *T*Δ*S*° and Δ*H*° (Figure 7A). The plots of *T*Δ*S*° versus Δ*H*° for these sequences in the absence of liposome also display linear relationships (Figure 7B) (19,57,58). The slope of the plot for the G-quadruplex formation in the presence of liposome (0.85) is comparable with that observed for cyclodextrin host-guest complex formation (0.88) (59), a protein–protein interaction (0.92) (60). The slopes of the plots for G-quadruplex formation in the absence of liposome (0.84) (19,57,58) and for duplex formation in the absence of liposome (0.86) (61) were similar and slightly higher than the slope for supramolecular complex formation (0.62) in the absence of liposome (62) (Table 3). The intercept of the *T*Δ*S*° versus Δ*H*° plot for supramolecular complex formation, cyclodextrin host-guest complex formation and a protein–protein interaction were +0.11, +2.87 and +10.6 kcal mol^−1^, respectively. In contrast, duplex (−0.30 kcal mol^−1^) and G-quadruplex (−3.45 and −2.70 kcal mol^−1^) formation resulted in negative intercepts. Because the intercept reflects the intrinsic complex stability, supramolecular complex formation, cyclodextrin host-guest complex formation and the protein–protein interaction are stabilized without additional enthalpy contributions, whereas nucleic acid structure formation requires enthalpic stabilization in the form of cation binding. The large negative intercept for the formation of G-quadruplexes reflects cation binding to the G-quartet cores of G-quadruplexes.

**Table 3.** Slope and intercept of enthalpy–entropy compensation plots for various molecular events

Molecular event	Slope	Intercept (kcal mol^−1^)
Supramolecular complex formation^a^	0.62	+0.11
Cyclodextrin host-guest complex formation^b^	0.88	+2.87
Protein–protein interaction^c^	0.92	+10.6
Nucleic acid duplex formation in the absence of liposome^d^	0.86	−0.30
G-quadruplex formation in the absence of liposome^e,f,g^	0.84	−3.45
G-quadruplex formation in the presence of liposome^h^	0.85	−2.70

^a, b, c, d, e, f, and g^See (53,50,51,52,21,48 and 49), respectively.

^h^This study.

To understand the difference between the negative intercept of the *T*Δ*S*° versus Δ*H*° plot for the G-quadruplex formation at the liposome surface compared with that in the bulk solution, we need to know the thermodynamic contribution of counter-ions bound per poly-ion charge (*ψ*). *ψ* can be expressed as a combination of condensation (c) and screening (sc) (63):

\begin{equation*} \psi = \psi _{\rm c} + \psi _{{\rm sc}} = 1 - (2\xi )^{ - 1} \end{equation*}

where *ξ* = *e*^2^/(*εkTb*), where *e* is the electronic charge magnitude, *ε* is the dielectric constant, *k* is Boltzmann's constant, *T* is the absolute temperature and *b* is the inter-phosphate distance. Therefore, *ψ* = *f* (*ε*,*b*) at constant temperature and, because nucleic acid structure formation occurs in the same dielectric medium,

\begin{equation*} \Delta \psi = - C\varepsilon \Delta b \end{equation*}

where Δ*ψ* = *ψ*_s_ − *ψ*_u_ and *ψ*_s_ and *ψ*_u_ are the thermodynamic contributions of counter-ions bound per poly-ion charge in the structured and unstructured states, respectively. *C* = *kT*/2*e*^2^ and Δ*b* = *b*_s_ − *b*_u_. *b*_s_ and *b*_u_ are inter-phosphate distances in the structured and unstructured states, respectively. Therefore, the small differences in the negative intercepts of the *T*Δ*S*° versus Δ*H*° plot for the formation of G-quadruplexes at the liposome surface compared with bulk solution result from changes in Δ*b*. In this case, in the unstructured state at the liposome surface the inter-phosphate distance is extended relative to that in bulk solution (Supplementary Figure S4B and S4B). A previous analysis showed that DNA transitioned from a folded state in the aqueous phase to a coil state on the phosphatidylethanolamine membrane (4). The extended structure may facilitate a biological reaction, such as transcription. The 0.75 kcal mol^−1^ difference in the negative intercepts for the formation of G-quadruplexes at the liposome surface compared with bulk solution is likely due to screening by lipid head groups; this indicates that fewer cations are bound upon G-quadruplex formation at the liposome surface than in bulk solution.

### Biological significance of the destabilization of G-quadruplexes at the liposome surface

There is much debate about the existence of G-quadruplexes in cells, although G-quadruplexes have been recently detected in human cells using a structure-specific antibody (64). We previously showed that the histone H3 tail and mimics of nuclear confinement can stabilize G-quadruplexes (23,24). Inside the eukaryotic cell, genomic DNA wraps around histone octamers to form nucleosomes, which are further organized to form the higher-order structure of chromatin (65–68). In condensed regions of chromatin, known as heterochromatin, attached to NM, gene expression is suppressed (12). Heterochromatin mainly consists of genetically inactive repetitive DNA sequences but becomes transcriptionally active under certain conditions (69). For example, telomeres, which are able to adopt G-quadruplex structures, are transcriptionally active under certain conditions (70). The formation of G-quadruplexes inhibits transcription *in vitro* (71), but localization to the NM may promote the unfolding of the G-quadruplexes, facilitating transcription. Some mRNAs are translated at the ER surface (12). Phosphatidylcholine accounts for more than 50% of total phospholipid content in NM and ER (26). Here we found that liposomes that mimic membrane surfaces destabilize G-quadruplexes. Therefore, chromatin function and gene expression could be regulated by alteration of the stability of DNA G–quadruplexes by organelle membranes.

We propose that mRNAs encoding membrane proteins form stable G-quadruplexes in the cytosol that prevent translation. When transported to the membrane surface, G–quadruplex structures are destabilized, allowing protein synthesis. Liposomes have been used as models for primitive cellular systems (72,73). Encapsulation of transcription and translation mixtures inside liposomes allow protein production, whereas no reaction occurs in liposome-free solution (74). Membranes likely influence transcription and translation efficiency by altering the structural stability of DNA and RNA. It has been reported that 20–30% of all genes in most genomes encodes membrane proteins (75). The translation of membrane proteins occurs at the ER membrane surface, and translated proteins interact with the ER. In mRNAs with G-rich regions that encode membrane proteins, the membrane surface may facilitate translation via the dissociation of G-quadruplexes. Membrane proteins represent over 50% of all recent pharmaceutical targets (76,77), and cell-free synthesis of membrane proteins within model membrane systems has been used to study these proteins. Rapid evolution of a wide range of membrane proteins using liposomes has been recently reported (78,79). Liposomes are very simple cell models that provide valuable information on key physical and chemical processes inside cells. As shown in this study, transcription was more efficient at the liposome surface than in solution when the template DNA contained a G-quadruplex-forming sequence, although the G-quadruplex is known to be an inhibitor of transcription.

In summary, we investigated the structures and stabilities of nucleic acid duplexes and G-quadruplexes in the presence of liposomes that mimic the NM, rough ER and IM. Our experimental findings revealed that the thermal stability of nucleic acid structures depends on the location of the molecules with respect to a liposome surface. The stabilities of G-quadruplexes decreased markedly when they were located at the liposome surface rather than in solution with liposomes. Such destabilization of G-quadruplexes originates from the unfavourable binding of the lipid head group to the guanine bases of DNA and RNA. Our results will guide studies of the stability of nucleic acid structures in conditions mimicking those *in vivo* and also be useful in development of *in vitro* gene expression systems.

## SUPPLEMENTARY DATA

Supplementary Data are available at NAR Online.

SUPPLEMENTARY DATA

## References

[1] Reddy K.L., Zullo J.M., Bertolino E., Singh H. (2008). Transcriptional repression mediated by repositioning of genes to the nuclear lamina. Nature.

[2] Martin F., MacDonald R. (1974). Liposomes can mimic virus membranes. Nature.

[3] Xia Y., Peng L. (2013). Photoactivatable lipid probes for studying biomembranes by photoaffinity labeling. Chem. Rev..

[4] Tsuji A., Yoshikawa K. (2010). ON-OFF switching of transcriptional activity of large DNA through a conformational transition in cooperation with phospholipid membrane. J. Am. Chem. Soc..

[5] Kurihara K., Tamura M., Shohda K., Toyota T., Suzuki K., Sugawara T. (2011). Self-reproduction of supramolecular giant vesicles combined with the amplification of encapsulated DNA. Nat. Chem..

[6] Noireaux V., Libchaber A. (2004). A vesicle bioreactor as a step toward an artificial cell assembly. Proc. Natl. Acad. Sci. U.S.A..

[7] Suga K., Tanabe T., Tomita H., Shimanouchi T., Umakoshi H. (2011). Conformational change of single-stranded RNAs induced by liposome binding. Nucleic Acids Res..

[8] Umakoshi H., Suga K., Bui H.T., Nishida M., Shimanouchi T., Kuboi R. (2009). Charged liposome affects the translation and folding steps of in vitro expression of green fluorescent protein. J. Biosci. Bioeng..

[9] Tang J., Signarvic R.S., DeGrado W.F., Gai F. (2007). Role of helix nucleation in the kinetics of binding of mastoparan X to phospholipid bilayers. Biochemistry.

[10] van den Bogaart G., Holt M.G., Bunt G., Riedel D., Wouters F.S., Jahn R. (2010). One SNARE complex is sufficient for membrane fusion. Nat. Struct. Mol. Biol..

[11] Wang L., Sigworth F.J. (2009). Structure of the BK potassium channel in a lipid membrane from electron cryomicroscopy. Nature.

[12] Lodish H., Berk A., Kaiser C.A., Krieger M., Scott M.P., Bretscher A., Ploegh H., Matsudaira P. (2008). Molecular Cell Biology.

[13] Heddi B., Phan A.T. (2011). Structure of human telomeric DNA in crowded solution. J. Am. Chem. Soc..

[14] Mukundan V.T., Phan A.T. (2013). Bulges in G-quadruplexes: broadening the definition of G-quadruplex-forming sequences. J. Am. Chem. Soc..

[15] Phan A.T., Kuryavyi V., Patel D.J. (2006). DNA architecture: from G to Z. Curr. Opin. Struct. Biol..

[16] Trajkovski M., da Silva M.W., Plavec J. (2012). Unique structural features of interconverting monomeric and dimeric G-quadruplexes adopted by a sequence from the intron of the N-myc gene. J. Am. Chem. Soc..

[17] Belotserkovskii B.P., Liu R., Tornaletti S., Krasilnikova M.M., Mirkin S.M., Hanawalt P.C. (2010). Mechanisms and implications of transcription blockage by guanine-rich DNA sequences. Proc. Natl. Acad. Sci. U.S.A..

[18] Bugaut A., Balasubramanian S. (2012). 5′-UTR RNA G-quadruplexes: translation regulation and targeting. Nucleic. Acids. Res..

[19] Miyoshi D., Karimata H., Sugimoto N. (2006). Hydration regulates thermodynamics of G-quadruplex formation under molecular crowding conditions. J. Am. Chem. Soc..

[20] Miyoshi D., Nakamura K., Tateishi-Karimata H., Ohmichi T., Sugimoto N. (2009). Hydration of Watson-Crick base pairs and dehydration of Hoogsteen base pairs inducing structural polymorphism under molecular crowding conditions. J. Am. Chem. Soc..

[21] Nakano S., Karimata H., Ohmichi T., Kawakami J., Sugimoto N. (2004). The effect of molecular crowding with nucleotide length and cosolute structure on DNA duplex stability. J. Am. Chem. Soc..

[22] Pramanik S., Nagatoishi S., Saxena S., Bhattacharyya J., Sugimoto N. (2011). Conformational flexibility influences degree of hydration of nucleic acid hybrids. J. Phys. Chem. B.

[23] Pramanik S., Nakamura K., Usui K., Nakano S., Saxena S., Matsui J., Miyoshi D., Sugimoto N. (2011). Thermodynamic stability of Hoogsteen and Watson-Crick base pairs in the presence of histone H3-mimicking peptide. Chem. Commun..

[24] Pramanik S., Nagatoishi S., Sugimoto N. (2012). DNA tetraplex structure formation from human telomeric repeat motif (TTAGGG):(CCCTAA) in nanocavity water pools of reverse micelles. Chem. Commun..

[25] Endoh T., Kawasaki Y., Sugimoto N. (2013). Suppression of gene expression by G-quadruplexes in open reading frames depends on G-quadruplex stability. Angew. Chem. Int. Ed. Engl..

[26] Vance D.E.V., Jean E.V. (2006). Biochemistry of Lipids, Lipoproteins and Membranes.

[27] Fasman G.D. (1975). Handbook of Biochemistry and Molecular Biology.

[28] Banchelli M., Betti F., Berti D., Caminati G., Bombelli F.B., Brown T., Wilhelmsson L.M., Norden B., Baglioni P. (2008). Phospholipid membranes decorated by cholesterol-based oligonucleotides as soft hybrid nanostructures. J. Phys. Chem. B.

[29] Kurz A., Bunge A., Windeck A.K., Rost M., Flasche W., Arbuzova A., Strohbach D., Muller S., Liebscher J., Huster D. (2006). Lipid-anchored oligonucleotides for stable double-helix formation in distinct membrane domains. Angew. Chem. Int. Ed. Engl..

[30] Mason J.T., Huang C., Biltonen R.L. (1983). Effect of liposomal size on the calorimetric behavior of mixed-chain phosphatidylcholine bilayer dispersions. Biochemistry.

[31] Koynova R., Caffrey M. (1998). Phases and phase transitions of the phosphatidylcholines. Biochim. Biophys. Acta.

[32] Chiu M.H., Prenner E.J. (2011). Differential scanning calorimetry: an invaluable tool for a detailed thermodynamic characterization of macromolecules and their interactions. J. Pharm. Bioallied Sci..

[33] Davis P.J., Fleming B.D., Coolbear K.P., Keough K.M. (1981). Gel to liquid-crystalline transition temperatures of water dispersions of two pairs of positional isomers of unsaturated mixed-acid phosphatidylcholines. Biochemistry.

[34] Ceppi P., Colombo S., Francolini M., Raimondo F., Borgese N., Masserini M. (2005). Two tail-anchored protein variants, differing in transmembrane domain length and intracellular sorting, interact differently with lipids. Proc. Natl. Acad. Sci. U.S.A..

[35] Ganesan M.G., Schwinke D.L., Weiner N. (1982). Effect of Ca2+ on thermotropic properties of saturated phosphatidylcholine liposomes. Biochim. Biophys. Acta.

[36] Augustynska D., Jemiola-Rzeminska M., Burda K., Strzalka K. (2012). Atomic force microscopy studies of the adhesive properties of DPPC vesicles containing beta-carotene. Acta Biochim. Pol..

[37] Berova N., Nakanishi K., Woody R.W. (2000). Circular Dichroism: Principles and Applications.

[38] Bloomfield V.A.C., Tinoco D.M. (2000). Nucleic Acids Structures, Properties, and Functions.

[39] Kypr J., Kejnovska I., Renciuk D., Vorlickova M. (2009). Circular dichroism and conformational polymorphism of DNA. Nucleic Acids Res..

[40] Tateishi-Karimata H., Sugimoto N. (2012). A-T base pairs are more stable than G-C base pairs in a hydrated ionic liquid. Angew. Chem. Int. Ed. Engl..

[41] Nakano M., Tateishi-Karimata H., Tanaka S., Sugimoto N. (2014). Choline ion interactions with DNA atoms explain unique stabilization of A-T base pairs in DNA duplexes: a microscopic view. J. Phys. Chem. B.

[42] Broxson C., Beckett J., Tornaletti S. (2011). Transcription arrest by a G quadruplex forming-trinucleotide repeat sequence from the human c-myb gene. Biochemistry.

[43] Tateishi-Karimata H., Isono N., Sugimoto N. (2014). New insights into transcription fidelity: thermal stability of non-canonical structures in template DNA regulates transcriptional arrest, pause, and slippage. PloS ONE.

[44] Janas T., Yarus M. (2006). Specific RNA binding to ordered phospholipid bilayers. Nucleic Acids Res..

[45] Michanek A., Kristen N., Hook F., Nylander T., Sparr E. (2010). RNA and DNA interactions with zwitterionic and charged lipid membranes - a DSC and QCM-D study. Biochim. Biophys. Acta.

[46] Michanek A., Yanez M., Wacklin H., Hughes A., Nylander T., Sparr E. (2012). RNA and DNA association to zwitterionic and charged monolayers at the air-liquid interface. Langmuir.

[47] Marty R., N'Soukpoe-Kossi C.N., Charbonneau D.M., Kreplak L., Tajmir-Riahi H.A. (2009). Structural characterization of cationic lipid-tRNA complexes. Nucleic Acids Res..

[48] Suga K., Umakoshi H., Tomita H., Tanabe T., Shimanouchi T., Kuboi R. (2010). Liposomes destabilize tRNA during heat stress. Biotechnol. J..

[49] Cevc G. (1990). Membrane electrostatics. Biochim. Biophys. Acta.

[50] Smirnov I.V., Shafer R.H. (2007). Electrostatics dominate quadruplex stability. Biopolymers.

[51] Neidle S., Balasubramanian S. (2006). Quadruplex Nucleic Acids.

[52] Atkins P.W. (1982). Physical Chemistry.

[53] Liu L., Guo Q.X. (2001). Isokinetic relationship, isoequilibrium relationship, and enthalpy-entropy compensation. Chem. Rev..

[54] Starikov E.B., Norden B. (2007). Enthalpy-entropy compensation: a phantom or something useful. J. Phys. Chem. B.

[55] Forrey C., Douglas J.F., Gilson M.K. (2012). The fundamental role of flexibility on the strength of molecular binding. Soft Matter.

[56] Lumry R., Rajender S. (1970). Enthalpy-entropy compensation phenomena in water solutions of proteins and small molecules: a ubiquitous property of water. Biopolymers.

[57] Olsen C.M., Lee H.T., Marky L.A. (2009). Unfolding thermodynamics of intramolecular G-quadruplexes: base sequence contributions of the loops. J. Phys. Chem. B.

[58] Tran P.L., Mergny J.L., Alberti P. (2011). Stability of telomeric G-quadruplexes. Nucleic Acids Res..

[59] Rekharsky M., Inoue Y. (2000). Chiral recognition thermodynamics of β-cyclodextrin: the thermodynamic origin of enantioselectivity and the enthalpy−entropy compensation effect. J. Am. Chem. Soc..

[60] De M., You C.C., Srivastava S., Rotello V.M. (2007). Biomimetic interactions of proteins with functionalized nanoparticles: a thermodynamic study. J. Am. Chem. Soc..

[61] Nakano S., Fujimoto M., Hara H., Sugimoto N. (1999). Nucleic acid duplex stability: influence of base composition on cation effects. Nucleic Acids Res..

[62] Hayashi T., Miyahara T., Koide N., Kato Y., Masuda H., Ogoshi H. (1997). Molecular recognition of ubiquinone analogues. specific interaction between quinone and functional porphyrin via multiple hydrogen bonds. J. Am. Chem. Soc..

[63] Record M.T., Lohman M.L., De Haseth P. (1976). Ion effects on ligand-nucleic acid interactions. J. Mol. Biol..

[64] Biffi G., Tannahill D., McCafferty J., Balasubramanian S. (2013). Quantitative visualization of DNA G-quadruplex structures in human cells. Nat. Chem..

[65] Arents G., Burlingame R.W., Wang B.C., Love W.E., Moudrianakis E.N. (1991). The nucleosomal core histone octamer at 3.1 A resolution: a tripartite protein assembly and a left-handed superhelix. Proc. Natl. Acad. Sci. U.S.A.

[66] Kornberg R.D. (1977). Structure of chromatin. Annu. Rev. Biochem..

[67] Luger K., Mader A.W., Richmond R.K., Sargent D.F., Richmond T.J. (1997). Crystal structure of the nucleosome core particle at 2.8 A resolution. Nature.

[68] Richmond T.J., Finch J.T., Rushton B., Rhodes D., Klug A. (1984). Structure of the nucleosome core particle at 7 A resolution. Nature.

[69] Oberdoerffer P., Sinclair D.A. (2007). The role of nuclear architecture in genomic instability and ageing. Nat. Rev. Mol. Cell Biol..

[70] Azzalin C.M., Reichenbach P., Khoriauli L., Giulotto E., Lingner J. (2007). Telomeric repeat containing RNA and RNA surveillance factors at mammalian chromosome ends. Science.

[71] Belotserkovskii B.P., Mirkin S.M., Hanawalt P.C. (2013). DNA sequences that interfere with transcription: implications for genome function and stability. Chem. Rev..

[72] Deamer D.W. (1985). Boundary structures are formed by organic components of the Murchison carbonaceous chondrite. Nature.

[73] Mansy S.S., Schrum J.P., Krishnamurthy M., Tobe S., Treco D.A., Szostak J.W. (2008). Template-directed synthesis of a genetic polymer in a model protocell. Nature.

[74] Stano P., D'Aguanno E., Bolz J., Fahr A., Luisi P.L. (2013). A remarkable self-organization process as the origin of primitive functional cells. Angew. Chem. Int. Ed. Engl..

[75] Krogh A., Larsson B., von Heijne G., Sonnhammer E.L. (2001). Predicting transmembrane protein topology with a hidden Markov model: application to complete genomes. J. Mol. Biol..

[76] Katzen F., Peterson T.C., Kudlicki W. (2009). Membrane protein expression: no cells required. Trends Biotechnol..

[77] Overington J.P., Al-Lazikani B., Hopkins A.L. (2006). How many drug targets are there. Nat. Rev. Drug Discov..

[78] Fujii S., Matsuura T., Sunami T., Kazuta Y., Yomo T. (2013). In vitro evolution of alpha-hemolysin using a liposome display. Proc. Natl. Acad. Sci. U.S.A..

[79] Stano P., Luisi P.L. (2013). Semi-synthetic minimal cells: origin and recent developments. Curr. Opin. Biotechnol..

