# Supplementary material for: Organelle-mimicking liposome dissociates G-quadruplexes and facilitates transcription
Source: Nucleic Acids Res. 2014 Oct 21;42(20):12949–59. doi: 10.1093/nar/gku998 (PMC4227800; doi:10.1093/nar/gku998)
Supplement: SUPPLEMENTARY DATA [file supp_42_20_12949__index.html]

Organelle-mimicking liposome dissociates G-quadruplexes and facilitates transcription — Organelle-mimicking liposome dissociates G-quadruplexes and facilitates transcription — SUPPLEMENTARY DATA 

# Organelle-mimicking liposome dissociates G-quadruplexes and facilitates transcription

## SUPPLEMENTARY DATA

**Files in this Data Supplement:**

- SUPPLEMENTARY DATA
