# Supplementary material for: Organelle-mimicking liposome dissociates G-quadruplexes and facilitates transcription
Source: Nucleic Acids Res. 2014 Oct 21;42(20):12949–59. doi: 10.1093/nar/gku998 (PMC4227800; doi:10.1093/nar/gku998)
Supplement: SUPPLEMENTARY DATA [file supp_gku998_nar-01757-f-2014-File002.pdf]

Organelle-Mimicking Liposome Dissociates G-quadruplexes and Facilitates Transcription

Smritimoy Pramanik,[1] Hisae Tateishi-Karimata,[1] and Naoki Sugimoto[1,2*]

[1]Frontier Institute for Biomolecular Engineering Research (FIBER), [2]Faculty of Frontiers of Innovative Research in Science and Technology (FIRST), Konan University, 7-1-20 Minatojima-minamimachi, Chuo-ku, Kobe 650-0047, Japan.

Page S1: Contents.

Page S2: Supplementary Table 1 for the thermodynamic parameters for the structural formations of mcG_n G-quadruplexes.

Page S3: Supplementary Table 2 for the change of thermodynamic parameters for formations of G-quadruplex by $1cG_n$

Page S4: Supplementary Table 3 for the thermodynamic parameters for the structural formations of $1crG_3$ G-quadruplex.

Page S5: Supplementary Table 4 for the thermodynamic parameters for the structural formations of $1cG_3$ G-quadruplexes.

Page S6: Supplementary Table 5 for the change of thermodynamic parameters for formations of G-quadruplex by $1cG_3$.

Page S7: Supplementary Figure 1 for schematic representation of the G-quadruplexes.

Page S8: Supplementary Figures 2 and 3 for the DLS and DSC of liposomes.

Page S9: Supplementary Figure 4 for the CD spectra and melting profiles of D and G_3.

Page S10: Supplementary Figure 5 for the CD spectra and melting profiles of G_2 and G_4.

Page S11: Supplementary Figure 6 for the CD spectra and melting profiles of $1cG_2$ and $1cG_4$.

Page S12: Supplementary Figure 6 for the CD spectra of $1cG_n$ in the absence and presence of POPC liposome.

Page S13: Supplementary Figures 8 and 9 for the CD spectra and melting profiles of $1cG_3$.

Page S14: Supplementary Figure 10 for the melting profiles of $1cG_2$ and $1cG_4$.

Page S15: Supplementary Figure 11 for the CD spectra of $1crG_3$ in the absence and presence of POPC liposome.

Page S16: Supplementary Figure 12 for the melting profiles of $1crG_3$.

Page S17: Supplementary Figures 13 and S14 for the CD spectra and melting profiles of $2cG_3$, $3cG_3$ and $4cG_3$.

Page S18: Supplementary Figure 15 for the denaturing gel electrophoresis of products of transcription reactions.

Supplementary Table 1. Thermodynamic parameters for formation of the G-quadruplex structures by mcG_n[a]

POPC Concentration (mM)	$\Delta H°$ (kcal mol^{-1})	$T\Delta S°$ (kcal mol^{-1})	$\Delta G°_{25}$ (kcal mol^{-1})	T_m (°C)
		**$1cG_2$**		
0.0	−30.7 ± 0.4	−29.3 ± 0.4	−1.4 ± 0.1	38.5
2.0	−26.6 ± 0.2	−25.7 ± 0.1	−0.9 ± 0.1	33.0
		**$1cG_4$**		
0.0	−62.1 ± 0.4	−54.7 ± 0.3	−7.4 ± 0.1	65.5
2.0	−38.1 ± 0.7	−33.9 ± 0.7	−4.2 ± 0.1	59.5
		**$2cG_3$**		
0.0	−43.1 ± 0.1	−40.2 ± 0.1	−2.9 ± 0.1	46.0
2.0	−27.8 ± 0.4	−26.4 ± 0.4	−1.4 ± 0.1	40.5
		**$3cG_3$**		
0.0	−40.2 ± 0.4	−37.9 ± 0.3	−2.3 ± 0.1	42.0
2.0	−31.7 ± 0.3	−30.3 ± 0.4	−1.4 ± 0.1	38.0
		**$4cG_3$**		
0.0	−43.3 ± 0.7	−41.1 ± 0.6	−2.2 ± 0.2	40.0
2.0	−33.1 ± 0.5	−31.8 ± 0.6	−1.3 ± 0.2	36.0

[a]All experiments were carried out in a buffer containing 10 mM sodium phosphate (pH 7.0), 10 mM NaCl, and 1 mM Na_2EDTA without or with 2.0 mM POPC. Values are means ± standard deviations from three independent measurements. Melting temperatures were measured at a total strand concentration of 20 μM.

Supplementary Table 2. Changes in thermodynamic parameters for formation of G-quadruplex by $1cG_n$ (n = 2, 3, and 4) in the presence of 2.0 mM POPC relative to its absence

**G-quadruplex**	**$\Delta\Delta H°$ (kcal mol^{-1})**	**$\Delta(T\Delta S°)$ (kcal mol^{-1})**	**$\Delta\Delta G°_{25}$ (kcal mol^{-1})**
$1cG_2$	+4.1	+3.6	+0.5
$1cG_3$	+17.9	+16.0	+1.9
$1cG_4$	+24.0	+20.8	+3.2

Supplementary Table 3. Thermodynamic parameters for the formation of G-quadruplex structure by $1crG_3$[a]

POPC Concentration (mM)	$\Delta H°$ (kcal mol^{-1})	$T\Delta S°$ (kcal mol^{-1})	$\Delta G°_{25}$ (kcal mol^{-1})	T_m (°C)
0.0	−45.8± 0.5	−40.6 ± 0.6	−5.2 ± 0.1	62.5
2.0	−23.5 ± 0.4	−22.0 ± 0.3	−1.5 ± 0.1	54.5

[a]All experiments were carried out in a buffer containing 10 mM sodium phosphate (pH 7.0), 10 mM NaCl, and 1 mM Na_2EDTA without or with 2.0 mM POPC. Values are means ± standard deviations from three independent measurements. Melting temperatures were measured at a total strand concentration of 20 µM.

Supplementary Table 4. Thermodynamic parameters for the formation of G-quadruplex structure by $1cG_3$[a]

**Liposome (2.0 mM)**	**$\Delta H°$ (kcal mol^{-1})**	**$T\Delta S°$ (kcal mol^{-1})**	**$\Delta G°_{25}$ (kcal mol^{-1})**	**T_m (°C)**
–	-40.0 ± 0.6	-36.9 ± 0.5	-3.1 ± 0.1	50.0
IM	-28.0 ± 0.5	-26.0 ± 0.4	-2.0 ± 0.1	47.0
NM	-24.9 ± 0.8	-23.4 ± 0.3	-1.5 ± 0.1	45.0
ER	-23.1 ± 0.3	-21.7 ± 0.2	-1.4 ± 0.1	42.5

[a]All experiments were carried out in a buffer containing 10 mM sodium phosphate (pH 7.0), 10 mM NaCl, and 1 mM Na_2EDTA without or with 2.0 mM liposome. Values are means ± standard deviations from three independent measurements. Melting temperatures were measured at a total strand concentration of 20 μM.

Supplementary Table 5. Changes of thermodynamic parameters of G-quadruplex formed by $1cG_3$ in the presence of IM, NM, and ER mimicking liposomes and POPC liposomes

**Liposome (2.0 mM)**	**Molar ratio of POPC (%)**	**$\Delta\Delta H°$ (kcal mol^{-1})**	**$\Delta(T\Delta S°)$ (kcal mol^{-1})**	**$\Delta\Delta G°_{25}$ (kcal mol^{-1})**
IM	43	+12.0	+10.9	+1.1
NM	69	+15.1	+13.5	+1.6
ER	75	+16.9	+15.2	+1.7
POPC	100	+17.9	+16.0	+1.9

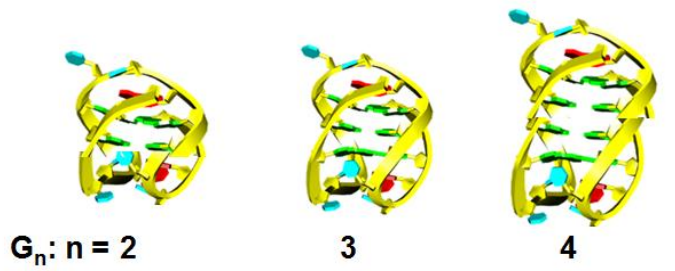

Supplementary Figure 1. Schematic representations of G_n (n = 2, 3, and 4) structures.

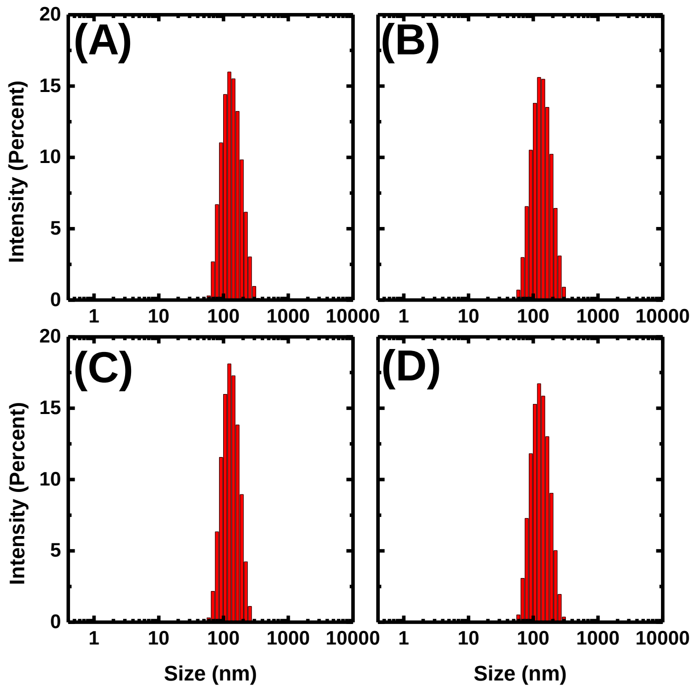

Supplementary Figure 2. Diameter distributions of **(A)** POPC, **(B)** IM, **(C)** NM, and **(D)** ER liposomes at 25 °C.

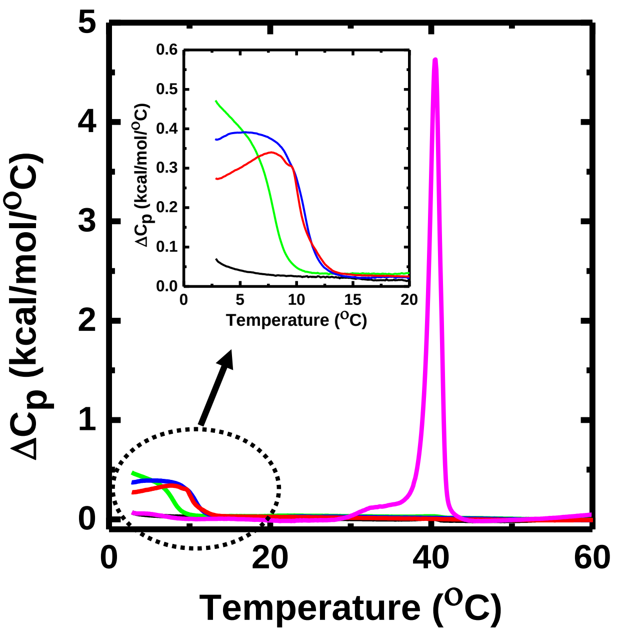

Supplementary Figure 3. DSC heating thermograms for POPC (black), IM (red), NM (blue), ER (green), and DPPC (pink) liposomes. The inset shows DSC profiles in the region from 0 to 20 °C.

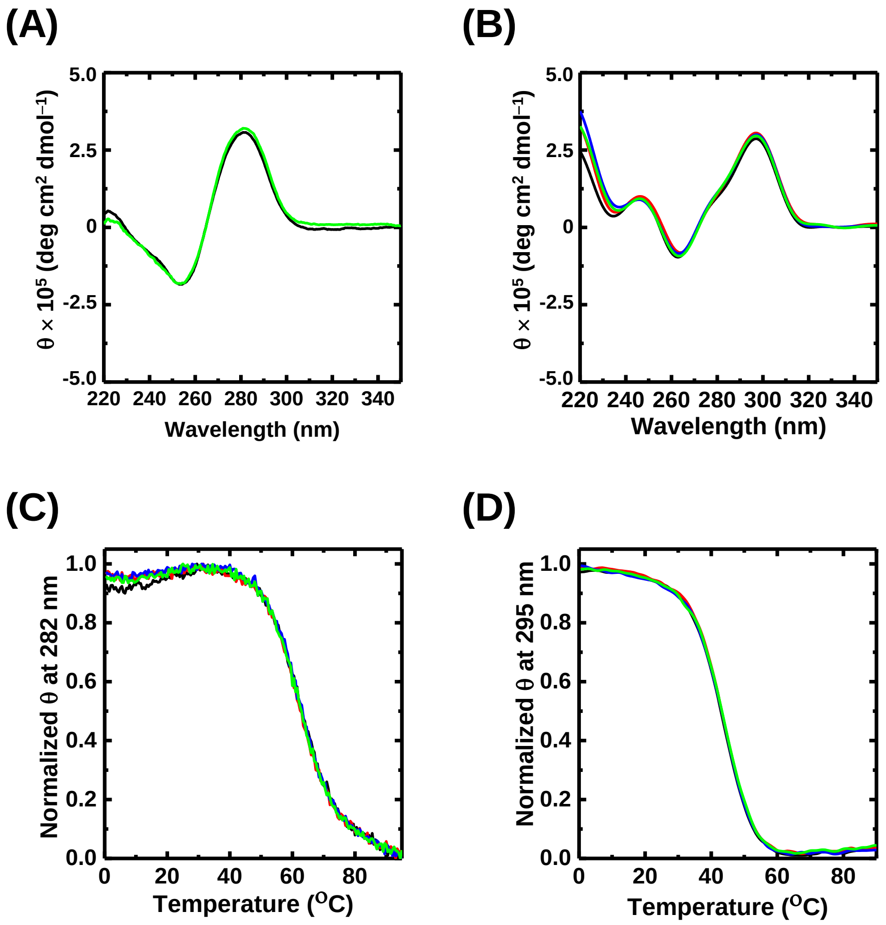

Supplementary Figure 4. CD spectra of 20 μM **(A)** D and **(B)** G_3 at 4 °C and normalized CD melting profiles of **(C)** D at 282 nm and **(D)** G_3 at 295 nm in the absence (black) and presence of 2.0 mM IM (red), NM (blue), and ER (green) liposomes.

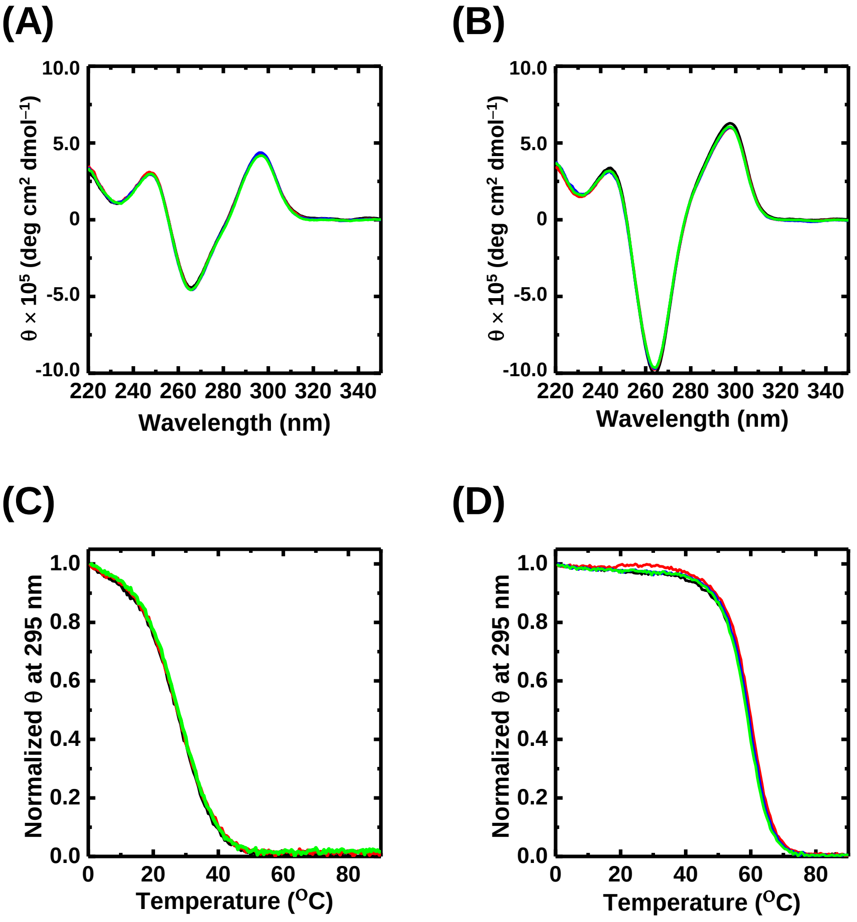

Supplementary Figure 5. CD spectra of 20 μM **(A)** G_2 and **(B)** G_4 at 4 °C and normalized CD melting profiles of **(C)** G_2 and **(D)** G_4 at 295 nm in the absence (black) and presence of 2.0 mM IM (red), NM (blue), and ER (green) liposomes.

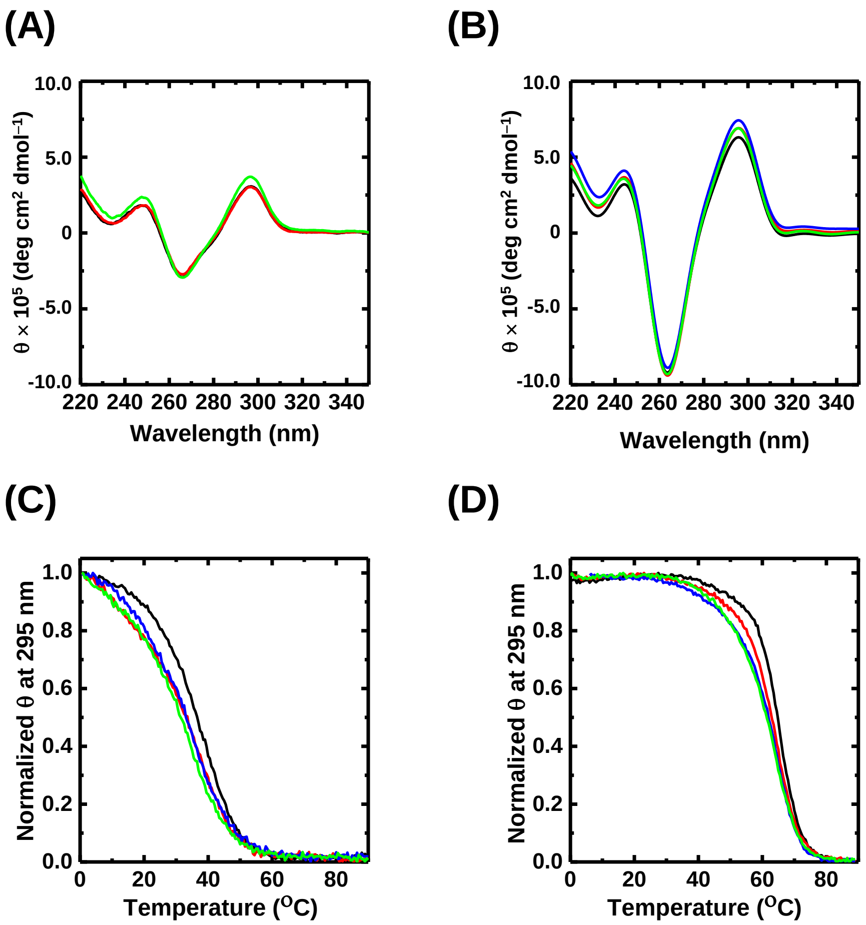

Supplementary Figure 6. CD spectra of 20 μM **(A)** 1cG$_2$ and **(B)** 1cG$_4$ at 4 °C and normalized CD melting profiles of **(C)** 1cG$_2$ and **(D)** 1cG$_4$ at 295 nm in the absence (black) and presence of 2.0 mM IM (red), NM (blue), and ER (green) liposomes.

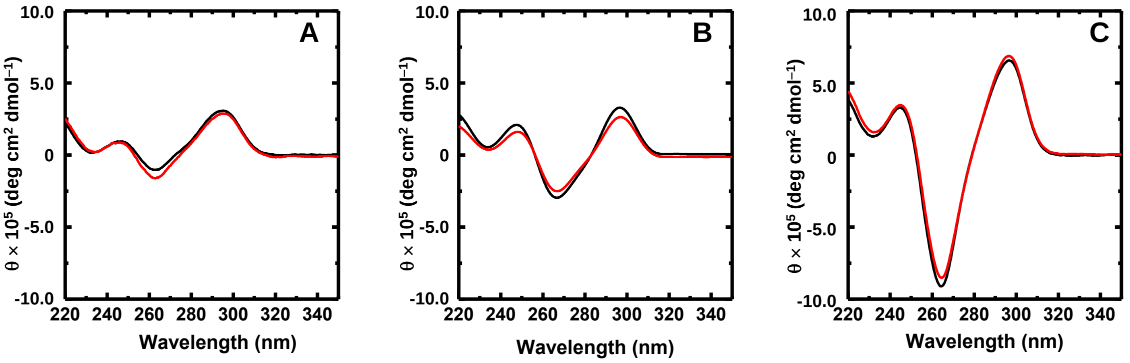

Supplementary Figure 7. CD spectra of 20 μM **(A)** 1cG$_3$, **(B)** 1cG$_2$, and **(C)** 1cG$_4$ in the absence (black) and presence (red) of 2.0 mM POPC liposomes at 4 °C.

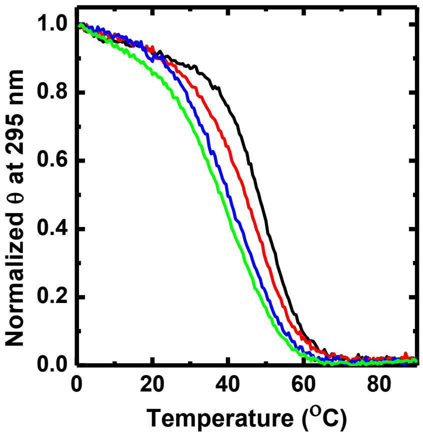

Supplementary Figure 8. Normalized CD melting profiles at 295 nm of 20 μM total strand concentration of 1cG_3 in the absence (black) and presence of 0.5 (red), 1.0 (blue), and 2.0 mM (green) POPC liposomes.

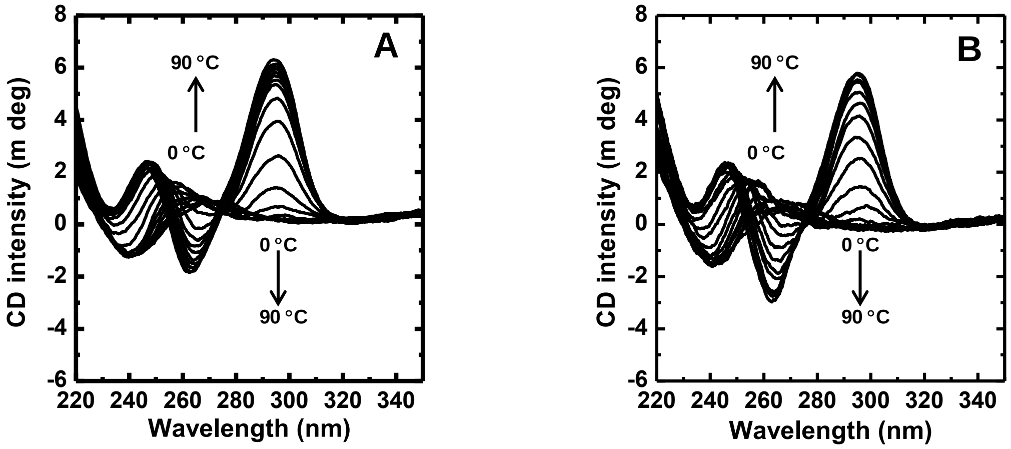

Supplementary Figure 9. CD spectra of 20 μM 1cG_3 in **(A)** the absence and **(B)** presence of 2.0 mM POPC liposomes at temperatures from 0 to 90 °C.

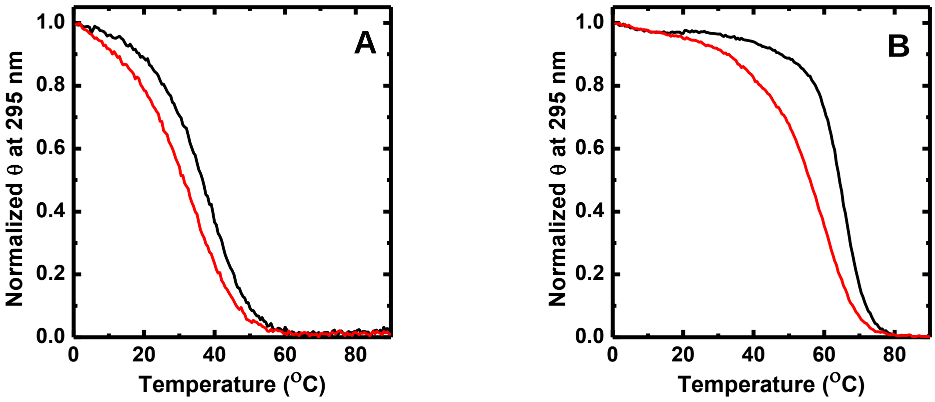

Supplementary Figure 10. Normalized CD melting profiles at 295 nm of 20 μM total strand concentration of **(A)** 1cG_2 and **(B)** 1cG_4 in the absence (black) and presence (red) of 2.0 mM POPC liposomes.

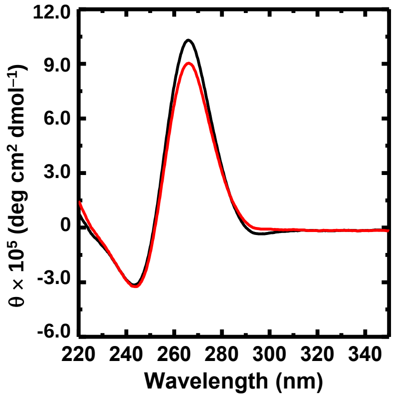

Supplementary Figure 11. CD spectra of 20 μM 1crG$_3$ in the absence (black) and presence (red) of 2 mM POPC liposomes at 4 °C.

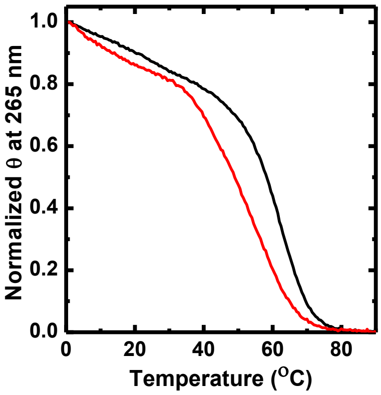

Supplementary Figure 12. Normalized CD melting profiles at 265 nm of 20 μM total strand concentration of 1crG$_3$ in the absence (black) and presence (red) of 2.0 mM POPC liposomes.

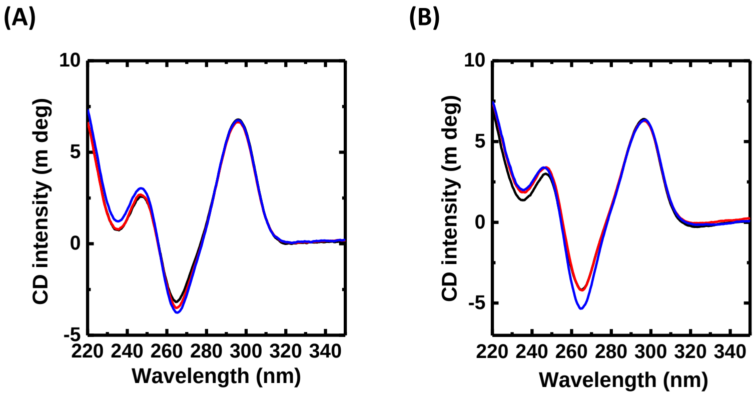

Supplementary Figure 13. CD spectra of 20 μM 2cG_3 (black), 3cG_3 (red), and 4cG_3 (blue) in **(A)** absence and **(B)** presence of 2.0 mM POPC liposomes at 4 °C.

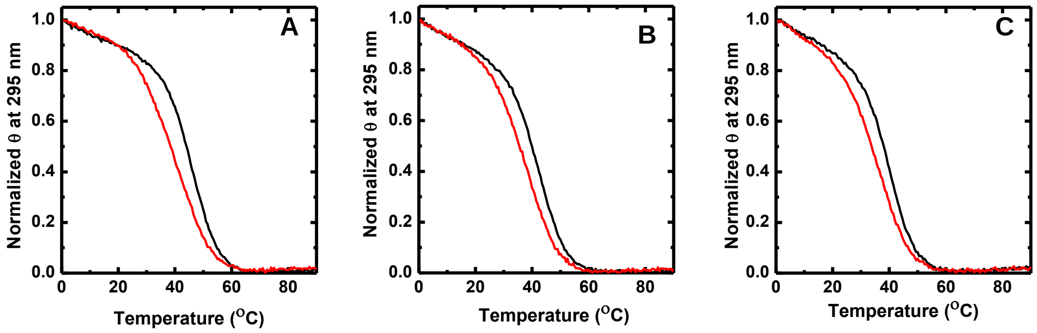

Supplementary Figure 14. Normalized CD melting profiles at 295 nm of 20 μM total strand concentration of **(A)** 2cG_3, **(B)** 3cG_3, and **(C)** 4cG_3 in the absence (black) and presence (red) of 2.0 mM POPC liposomes.

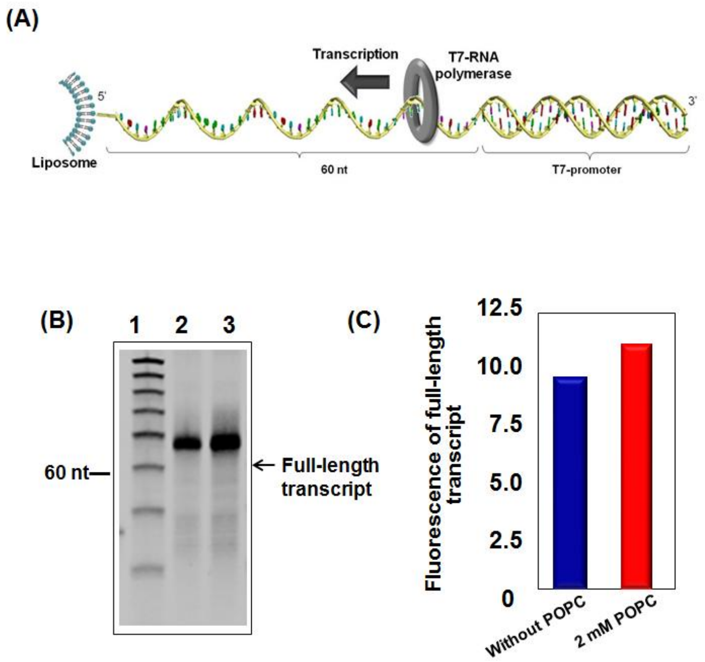

Supplementary Figure 15. Effects of membrane surface on transcription of a linear template DNA sequence. **(A)** Illustration of the template DNA immobilized on a liposome. **(B)** Denaturing gel electrophoresis of products of transcription reactions performed for 90 min at 37 °C. Reaction mixtures contained 0.3 μM T7 polymerase and 1.5 μM DNA template in a buffer containing 10 mM NaCl, 40 mM Tris-HCl (pH 8.0), 8 mM $MgCl_2$ and 2 mM spermidine in the absence and presence of 2.0 mM POPC. Lane 1, size marker; lane 2, transcription products in the absence of POPC; lane 3, transcription products in the presence of 2.0 mM POPC. **(C)** Comparison of fluorescence intensities of full-length transcripts from gel bands in panel **B**.
